# Cholesterol metabolic reprogramming mediates microglia-induced chronic neuroinflammation and hinders neurorestoration following stroke

**DOI:** 10.1038/s42255-025-01379-7

**Published:** 2025-09-23

**Authors:** Qiang Zhao, Jiajian Li, Jingjing Feng, Xin Wang, Yueting Liu, Fei Wang, Liang Liu, Bingxue Jin, Ming Lin, Ya-chao Wang, Xiuhua Guo, Jieli Chen, Junwei Hao

**Affiliations:** 1 Department of Neurology, Xuanwu Hospital Capital Medical University, National Center for Neurological Disorders, Beijing, China; 2 Department of Neurosurgery, The Institute of Translational Medicine, Shenzhen Second People’s Hospital/The First Affiliated Hospital of Shenzhen University, Shenzhen, China; 3 Department of Epidemiology and Health Statistics, School of Public Health, Capital Medical University, Beijing, China; 4 Department of Neurology, Tianjin Huanhu Hospital, Tianjin Key Laboratory of Cerebral Vascular and Neurodegenerative Diseases, Tianjin, China; 5 Beijing Municipal Geriatric Medical Research Center, Beijing, China; 6 Key Laboratory for Neurodegenerative Diseases of Ministry of Education, Beijing, China

**Keywords:** Stroke, Chronic inflammation, Homeostasis, Metabolism

## Abstract

Chronic neuroinflammation is a major obstacle to post-stroke recovery, yet the underlying mechanisms, particularly the link between prolonged microglial activation and cholesterol metabolism, are not fully known. Here we show that ischaemic injury induces persistent microglial activation that perpetuates chronic inflammation, leading to microglial cholesterol accumulation and metabolic reprogramming. Using single-cell RNA sequencing, we identified distinct stroke-associated foamy microglia clusters characterized by extensive reprogramming of cholesterol metabolism. Furthermore, direct intracerebral free cholesterol or cholesterol crystal infusion recapitulated sustained microglial activation, directly linking aberrant cholesterol metabolism to prolonged neuroinflammatory responses. Therapeutically, we demonstrate that reducing microglial cholesterol overload through genetic or pharmacological activation of CYP46A1 in male mice promotes white matter repair and functional recovery. These findings highlight microglial cholesterol metabolism as a key driver of post-stroke inflammation, offering therapeutic strategies targeting cholesterol metabolism to mitigate long-term brain damage and promote neurorestoration, potentially improving stroke-related disability outcomes.

## Abstract (web summary)

Persistent microglial activation upon ischaemic injury leads to the formation of stroke-associated foamy microglia, perpetuating long-term inflammation, white matter damage and functional impairments. These effects can be ameliorated by reducing microglial cholesterol overload through activation of CYP46A1.

## Main

Most patients with ischaemic stroke fail to receive thrombolytic treatment with recombinant tissue plasminogen activator because of its narrow therapeutic window, limited to 4.5 h after onset^1^. By contrast, during the chronic phase, a potentially broader therapeutic window may exist, offering opportunities to facilitate late-stage recovery and reduce stroke-related disabilities and recurrence. Therefore, targeting neurorestorative mechanisms in the chronic phase offers a promising approach to enhancing recovery and improving outcomes for stroke survivors.

Microglia, the primary resident immune cells of the central nervous system, have dual roles in the acute phase of ischaemic stroke^2^, exhibiting both protective and detrimental effects. For example, microglia rapidly migrate to the lesion site, where they exacerbate tissue damage by releasing inflammatory cytokines and cytotoxic substances. However, they also contribute to tissue repair and restructuring by clearing debris and generating anti-inflammatory cytokines following ischaemic injury^2^. In the chronic phase, abnormal microglial activation persists within the infarct and peri-infarct regions for weeks to months, as demonstrated in both clinical and experimental studies^3–5^. Despite these observations, a systematic characterization of these altered microglial states and the mechanisms sustaining prolonged activation during the chronic phase post stroke is lacking. Elucidating the role of chronically activated microglia and identifying targets to mitigate their prolonged activation could potentially reduce stroke recurrence and related disabilities.

The brain, as the most cholesterol-rich organ, contains about 25% of the body’s total cholesterol^6,7^. In the adult brain, approximately 70% of the cholesterol is localized within myelin sheaths, which are formed by oligodendrocytes to insulate axons^8^. Following ischaemic stroke, microglia engulf substantial amounts of cellular debris, particularly cholesterol, from the lesion area. However, it remains unclear whether this process alters cholesterol metabolism within microglia and whether such metabolic reprogramming affects microglial function during the chronic phase after stroke. Cholesterol in the brain is largely isolated from the rest of the body by the intact blood–brain barrier (BBB) and is synthesized exclusively through de novo pathways^8^. Nevertheless, cholesterol can be continuously exported from the brain by the cytochrome P450 family 46 subfamily A member 1 (CYP46A1) enzyme, which converts cholesterol into 24S-hydroxycholesterol. This metabolite can cross the BBB, enter the bloodstream and be metabolized in the liver^9^. Studies have shown that genetically increasing *Cyp46a1* expression in mice improves symptoms in models of Alzheimer’s disease, Huntington’s disease and Niemann–Pick disease type C^10–12^. Moreover, efavirenz (EFV), an anti-HIV medication that crosses the BBB, can activate CYP46A1 activity and increase *Cyp46a1* expression^13,14^. Notably, EFV has been demonstrated to improve behavioural performance in Alzheimer’s disease mouse models and to enhance brain cholesterol metabolism in human participants with early-stage Alzheimer’s disease, as evidenced in a clinical trial^10,15^.

In this study, we demonstrate significantly prolonged microglial activation and persistent neuroinflammation post stroke by immunohistochemistry and bulk RNA sequencing (RNA-seq). Transcriptomic analysis and cholesterol visualization revealed that experimental stroke induces cholesterol metabolic reprogramming in microglia during the chronic phase. Single-cell RNA sequencing (scRNA-seq) of microglia further identified a distinct population of stroke-associated foamy microglia (SAM-foamy) clusters within the brain parenchyma during the chronic stage. Notably, injection of cholesterol (either free cholesterol or cholesterol crystals) into brain parenchyma leads to sustained microglial activation, linking cholesterol deposition with persistent microglia-mediated neuroinflammation. Finally, we show that modulating *Cyp46a1* by genetic and pharmacological intervention reduces chronic neuroinflammation, highlighting a potential therapeutic approach for promoting recovery after ischaemic stroke in male mice.

## Results

### Characterizing the role of microglia in acute and chronic neuroinflammation post ischaemic stroke in male mice

**Fig. 1:**
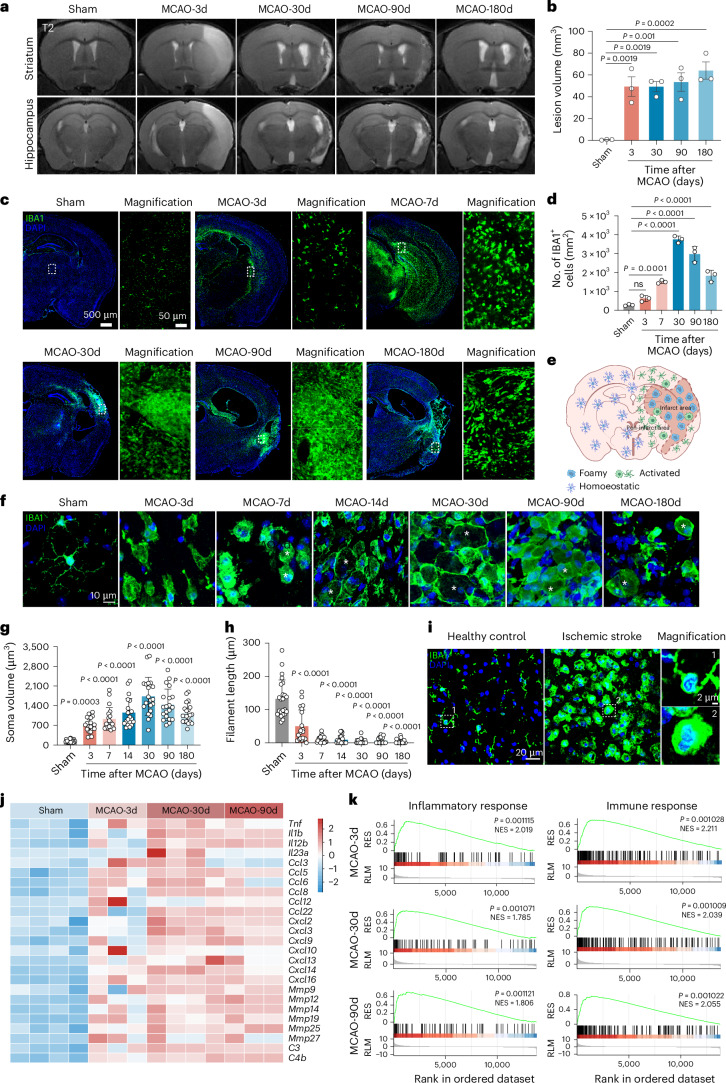
Microglia elicit a long-lasting brain-resident inflammatory response after MCAO. **a**,**b**, Representative T2-weighted MRI images (**a**) and quantification (**b**) of persistent ischaemic lesion quantified across the entire brain at acute and chronic stages after MCAO; *n* = 3 mice per group. **c**,IBA1 immunostaining reveal a significant increase in resident microglia at days 3, 7, 30, 90 and 180 after MCAO. For each image, the right panel shows a higher-magnification image of the lesion core (highlighted by the dashed white rectangle. **d**, Quantitative data of IBA1^+^ cell numbers in **c** across acute and chronic stages of MCAO; *n* = 3 mice per group. **e**, Schematic illustration of cerebral infarction and corresponding microglial morphological changes in the lesion core and peri-lesion regions. **f**, IBA1 immunostaining showing microglial morphology in the lesion area at days 3, 7, 14, 30, 90 and 180 post MCAO in male mice. Asterisks indicate foamy microglia. **g**,**h**, Quantification of morphological parameters, including soma volume (**g**) and filament length (**h**), is shown for MCAO-3d, MCAO-7d, MCAO-14d, MCAO-30d, MCAO-90d and MCAO-180d (*n* = 20 cells from three mice each) and sham (*n* = 23 cells from three mice). **i**, IBA1 immunostaining of human brain samples from a patient with ischaemic stroke (*n* = 1) and a healthy control (*n* = 1). Dashed white squares indicate regions shown at higher magnification. **j**, Heatmap showing upregulation of inflammatory genes in microglia isolated at 3, 30 and 90 days post MCAO compared to sham control; *n* = 4 mice per group for sham and MCAO-30d groups; *n* = 3 mice per group for MCAO-3d and MCAO-90d groups. **k**, GSEA showing upregulation of inflammatory and immune response pathways in microglia at both acute and chronic stages post MCAO. Normalized enrichment scores (NES) and *P* values are displayed; *n* = 4 mice per group for sham and MCAO-30d groups; *n* = 3 mice per group for MCAO-3d and MCAO-90d groups. RES, running enrichment score; RLM, ranked list metric. Data are mean and s.d. Statistical significance was assessed using one-way ANOVA (**b**,**d**,**g**,**h**) followed by Dunnett’s multiple comparisons test, or a one-sided permutation test, and the resulting *P* values were adjusted for multiple comparisons (**k**). Source data

**Extended Data Fig. 1:**
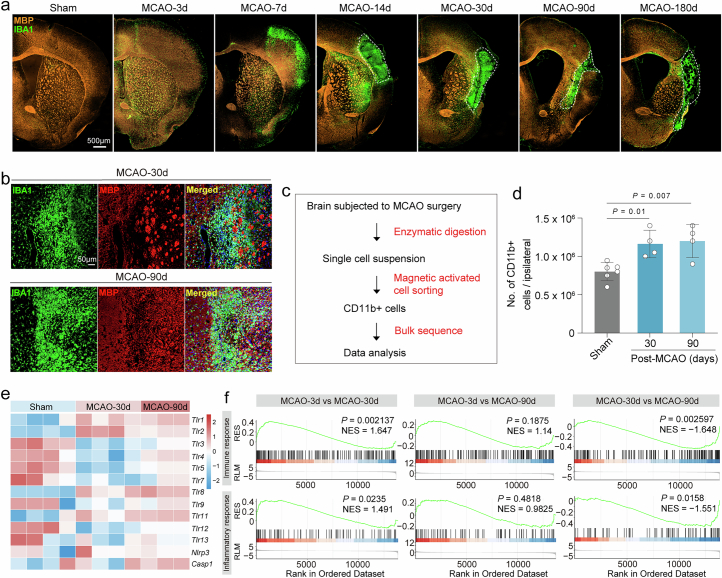
Microglia induce a sustained brain-resident inflammatory response after MCAO. **a**, Double immunostaining for IBA1 (microglial marker) and myelin basic protein (MBP, myelin marker) illustrates sustained myelin loss and microglial accumulation at days 3, 7, 14, 30, 90, and 180 post-MCAO. Dashed lines indicate regions of MBP disruption. Representative images from one of three independent experiments are shown. Scale bar, 500 µm. **b**, Co-immunostaining of MBP and IBA1 at days 30 and 90 post-MCAO shows spatial association between microglial activation and regions of myelin damage. Representative images from one of three independent experiments are shown. Scale bar, 50 µm. **c**, Workflow of bulk RNA-seq analysis, detailing the collection and processing of CD11b^+^ microglia for transcriptomic profiling post-MCAO. **d**, Quantification of CD11b^+^ cells reveals increased microglia number during the chronic stage post-MCAO. sham, n = 6 mice/group; MCAO group, n = 4 mice/group. **e**, Heatmap illustrating changes of toll-like receptor (TLR) signaling and NOD-, LRR-, and pyrin domain-containing protein 3 (NLRP3) inflammasome pathways in microglia during the chronic stage post-MCAO. n = 4 mice for the sham group; n = 3 mice per MCAO group. **f**, Gene set enrichment analysis (GSEA) identifies positive enrichment of inflammatory and immune response pathways. Normalized enrichment scores (NES) and *P*-value for each gene set are displayed. n = 4 mice for the sham group; n = 3 mice per MCAO group. RES: Running enrichment score; RLM: Ranked list metric. Data are presented as mean ± SD. Statistical significance was assessed by one-way ANOVA (**d**) followed by Dunnett’s multiple comparisons test, or a one-sided permutation test, and the resulting P-values were adjusted for multiple comparisons(**f**). Source data

Post-stroke brain pathology is characterized by persistent and progressive deficits. T2-weighted MRI analyses (Fig. 1a,b) revealed persistent, widespread brain lesions, accompanied by significant myelin damage at multiple time points, including days 14, 30, 90 and 180 after middle cerebral artery occlusion (MCAO) (Extended Data Fig. 1a,b). Immunohistochemical analysis using ionized calcium-binding adaptor molecule 1 (IBA1, a microglia marker) demonstrated substantial microglial accumulation and prolonged activation in the lesion areas at 3, 7, 30, 90 and 180 days after MCAO (Fig. 1c,d). A schematic model of cerebral infarction (Fig. 1e) highlights the morphological states of microglia within the lesion regions. IBA1 immunostaining revealed distinct reactive microglial phenotypes, including lipid-laden ‘foamy’ microglia and hypertrophic ‘amoeboid’ microglia, highlighting dynamic morphological transitions across the acute and chronic phase (Fig. 1f–h). Quantitative analyses confirmed significant alterations in IBA1^+^ cell soma volume and filament length during both the acute and chronic phase (Fig. 1f–h), providing strong evidence of microglial activation and remodelling post stroke. Notably, activated microglia clustered around areas of damaged myelin, potentially exacerbating myelin injury and impairing white matter repair (Extended Data Fig. 1e). Importantly, parallel examination of human ischaemic stroke brain sections revealed morphologic changes and accumulation of microglia within the lesion cores (Fig. 1i).

Microglial activation involves not only morphological transformation but also increased proliferation and polarization. To examine these dynamics, we isolated CD11b^+^ microglia from the ipsilateral hemispheres of male mice post MCAO on days 30 and 90 using magnetic-activated cell sorting (MACS) (Extended Data Fig. 1c). Cell counting following MACS revealed a significant increase in microglial numbers during the chronic phase, indicating pronounced microglial proliferation at later stages of stroke (Extended Data Fig. 1d). Bulk RNA-seq of these isolated microglia revealed substantial upregulation of inflammatory genes, including *Tnf*, *Il1b*, *Il12b*, *Ccl5*, *Ccl8*, *Ccl22*, *Cxcl2*, *Cxcl3*, *Cxcl9*, *Cxcl10*, *Cxcl13*, *Cxcl14*, *Cxcl16*, *Mmp9*, *Mmp12*, *Mmp14*, *Mmp19*, *Mmp25*, *C3* and *C4b* on days 30 and 90 after stroke compared to sham controls (Fig. 1j). These findings highlight sustained microglial inflammatory programming during chronic post-stroke phases, suggesting a key role in long-term neuroinflammatory remodelling.

Gene set enrichment analysis (GSEA) revealed that CD11b⁺ microglia isolated from male mice at 3, 30 and 90 days post stroke exhibited significant enrichment of inflammatory and immune response-related gene sets compared to sham controls (Fig. 1k), with detailed normalized enrichment scores and *P* values reported for each pathway. Moreover, GSEA demonstrated that microglia from mice at 30 and 90 days post MCAO showed upregulated pathways associated with inflammatory and immune responses relative to the acute post-stroke phase (Extended Data Fig. 1g). Notably, the enrichment of these pathways declined slightly at 90 days compared to 30 days post MCAO, suggesting a gradual shift in microglial transcriptional activity over time (Extended Data Fig. 1g).

Although previous studies have indicated that cholesterol accumulation in macrophages triggers pro-inflammatory responses by enhancing Toll-like receptor (TLR) signalling^16^ and activating the NOD-like receptor family pyrin domain-containing protein 3 (NLRP3) inflammasome pathway^17^, our analysis of post-stroke microglia revealed minimal changes in NLRP3 inflammasome activity and TLR signalling pathways, including TLR3 and TLR4, with the notable exception of an upregulation in TLR8 (Extended Data Fig. 1f).

**Extended Data Fig. 2:**
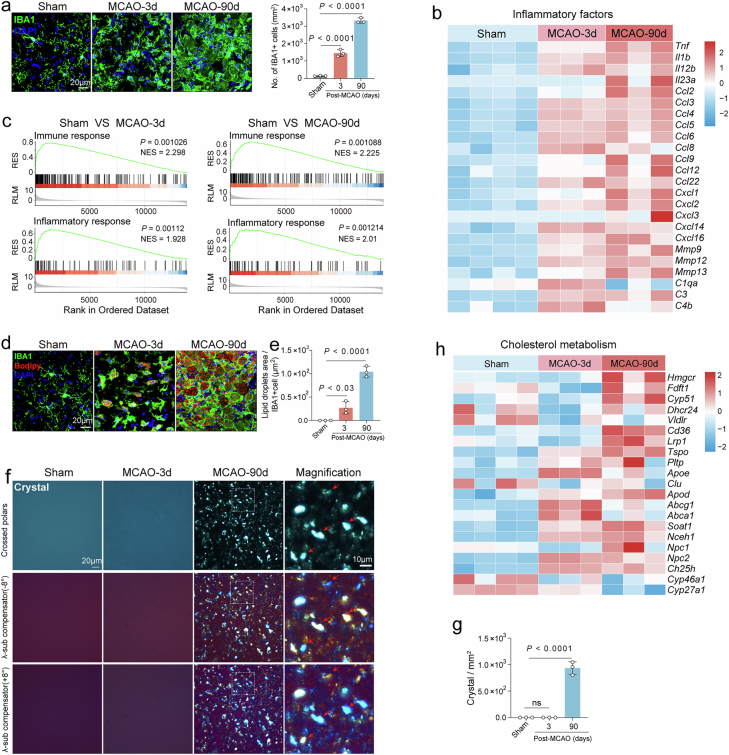
MCAO induces chronic neuroinflammation and microglial cholesterol metabolic reprogramming in female mice. **a**, IBA1 immunostaining showing altered microglial morphology in the lesion area at days 3 and 90 post-MCAO compared to sham controls in female mice. Scale bar, 20 µm; n = 3 mice/group; Representative images from one of two independent experiments are shown. **b**, Heatmap depicting upregulation of inflammatory genes in microglia isolated at days 3 and 90 post-MCAO compared to sham controls in female mice. n = 4 mice for the sham group; n = 3 mice per MCAO group. **c**, Gene set enrichment analysis (GSEA) identifying enrichment of inflammatory and immune-related pathways. Normalized enrichment scores (NES) and *P*-value for each gene set are displayed. sham, n = 4 mice/group; MCAO group, n = 3 mice/group. RES: Running enrichment score, RLM: Ranked list metric. **d**, **e**, Immunofluorescence detection of lipid droplets in microglia using BODIPY and IBA1 co-staining at 3 and 90 days post-MCAO in female mice, highlighting lipid accumulation over time. Scale bar, 20 µm; n = 3 mice/group; **f, g**, Polarized light microscopy (PLM) assessing cholesterol crystals at 3 and 90 days post-MCAO in female mice, demonstrating cholesterol crystal accumulation during the chronic phase. Crystal birefringence was confirmed by orientation-dependent interference colors under PLM with a first-order λ compensator. Main image: scale bar, 20 µm; Higher-magnification view: scale bar, 10 μm. n = 3 mice/group; ns: no significance; Representative images from one of three independent experiments are shown. **h**, Heatmap illustrating differential expression of cholesterol metabolism-associated genes in microglia at 3 and 90 days post-MCAO in female mice, revealing metabolic reprogramming. n = 4 mice for the sham group; n = 3 mice per MCAO group. Data are presented as mean ± SD. Statistical significance was assessed by one-way ANOVA (**a**, **e**, **g**) followed by Dunnett’s multiple comparisons test, or a one-sided permutation test, and the resulting *P*-values were adjusted for multiple comparisons©. Source data

Furthermore, we observed sustained microglial activation in female mice following MCAO. IBA1 immunostaining showed an increased number of microglia in the brain at both 3 and 90 days after MCAO (Extended Data Fig. 2a). Bulk RNA-seq further revealed that microglia in female mice exhibited increased levels of pro-inflammatory factors during both the acute and chronic phases following MCAO (Extended Data Fig. 2b). Additionally, gene pathway enrichment analysis identified significant enrichment of the inflammatory and immune response pathway in microglia from female mice throughout these phases after MCAO (Extended Data Fig. 2c).

In summary, ischaemic stroke induces prolonged microglial activation and persistent neuroinflammation in both male and female mice, which probably exacerbates white matter damage and impairs neurological recovery.

### Sustained microglia depletion during the chronic stage post stroke ameliorates stroke-induced neurologic lesions in male mice

**Fig. 2:**
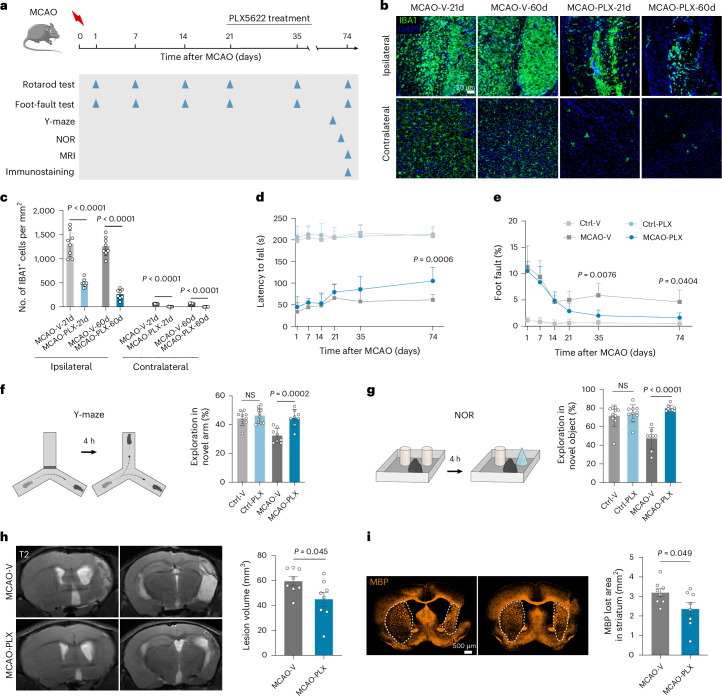
Continual microglial depletion during the chronic stage improves stroke-induced neurological outcomes. **a**, Experimental timeline showing PLX administration to MCAO male mice during the chronic stage (from 14 days to 74 days post MCAO). NOR, novel object recognition. **b**,**c**, IBA1 immunostaining (**b**) and quantification (**c**) demonstrating PLX-induced microglial depletion in both ipsilateral and contralateral hemispheres during the chronic phase post MCAO; *n* = 8 mice per group. V, vehicle. **d**,**e**, Motor and balance functions were assessed using rotarod (**d**) and foot-fault (**e**) tests in MCAO mice treated with PLX or vehicle; *n* = 8 mice per group. **f**,**g**, Cognitive performance evaluation using the Y-maze test (day 72) (**f**) and NOR test (day 70) (**g**) in MCAO mice treated with PLX or vehicle; *n* = 8 mice per group. **h**, Representative T2-weighted MRI images showing reduced brain lesion volume in PLX-treated MCAO mice compared to vehicle controls; *n* = 8 mice per group. **i**, MBP immunostaining revealing enhanced white matter repair (reduced MBP^+^ area loss) in the striatum of PLX-treated MCAO mice compared to vehicle controls; *n* = 8 mice per group. Data are mean and s.d. Statistical significance was assessed by two-way ANOVA followed by Bonferroni’s multiple comparisons test (**c**–**e**), one-way ANOVA followed by Bonferroni’s multiple comparisons test (**f**,**g**) or two-tailed unpaired Student’s *t*-test (**h**,**i**). NS, not significant. Source data

**Extended Data Fig. 3:**
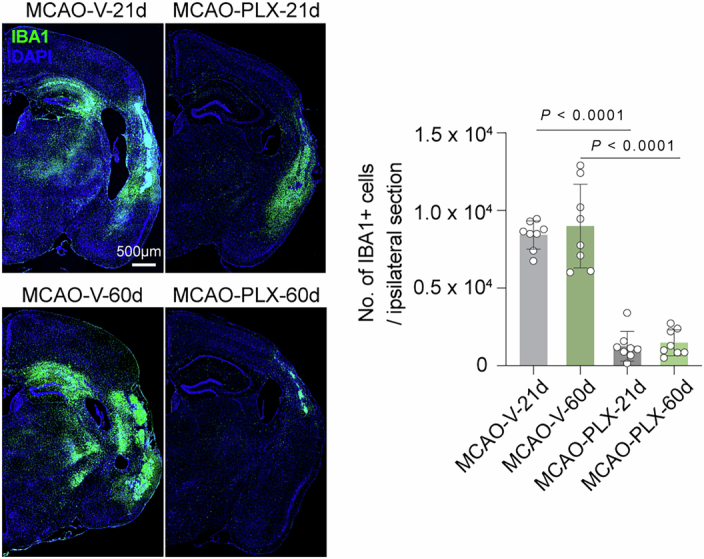
Effect of PLX-induced microglial depletion on ipsilateral microglia during the chronic phase post-MCAO. IBA1 immunostaining demonstrates a significant reduction in microglial presence following PLX5622 (PLX) administration during the chronic phase post-MCAO, confirming effective microglial depletion. Scale bar, 500 µm; n = 8 mice/group; Data are presented as mean ± SD. Statistical significance was assessed by two-way ANOVA followed by Bonferroni’s multiple comparisons test. Source data

To investigate the role of microglia during the chronic post-stroke phase, we selectively depleted microglia using PLX5622 (PLX) treatment (Fig. 2a). Microglial depletion efficiency was assessed in both the ipsilateral and contralateral hemispheres on days 21 and 60 following oral PLX administration. By both days 21 and 60, PLX significantly reduced IBA1^+^ microglia in the ipsilateral hemisphere (Fig. 2b,c and Extended Data Fig. 3). In the contralateral hemisphere, microglia numbers dropped from 66.63 mm^−2^ (vehicle control) to 2.68 mm^−2^ on day 60 following PLX treatment (Fig. 2b,c).

Motor function was evaluated using the rotarod and foot-fault tests. PLX-treated mice showed improved motor coordination, with significantly increased latency to fall on day 74 post MCAO (Fig. 2d) and fewer foot faults on days 35 and 74 (Fig. 2e) compared to vehicle-treated MCAO mice. Cognitive performance was assessed with the Y-maze and novel object recognition tests. PLX-treated MCAO mice spent more time exploring the novel arm and novel objects than vehicle-treated MCAO mice, indicating improved cognitive function (Fig. 2f,g).

MRI analysis revealed that PLX treatment reduced ipsilateral brain lesion volume at day 74 post MCAO compared to MCAO controls, as measured by T2-weighted imaging (Fig. 2h). Immunostaining for myelin basic protein (MBP) demonstrated enhanced white matter repair in PLX-treated MCAO mice, with significantly less MBP loss in the striatum at 74 days post stroke relative to untreated animals (Fig. 2i).

In summary, these findings indicate that sustained microglial depletion during the chronic phase post stroke promotes functional recovery and mitigates neurological deficits following ischaemic stroke.

### MCAO in male mice triggers cholesterol metabolic reprogramming in microglia during the chronic stage post stroke

**Extended Data Fig. 4:**
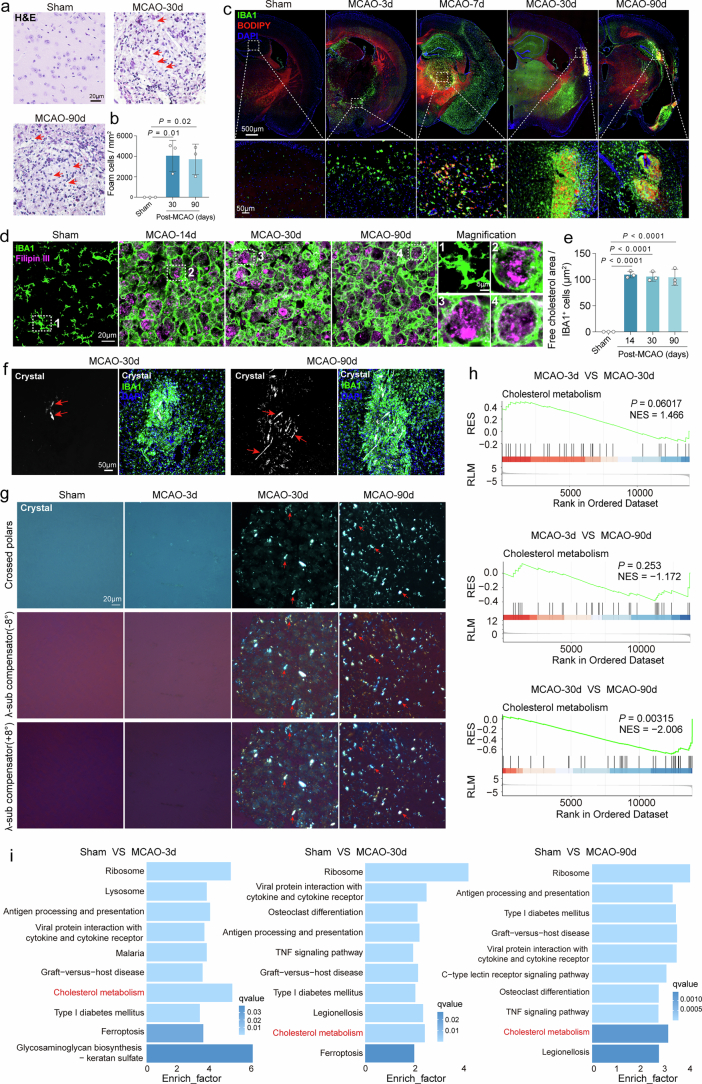
MCAO evokes cholesterol metabolic reprogramming in microglia during the chronic stage. **a, b**, H&E staining assessing foam cell formation in the infarct area during the chronic stage post-MCAO. Scale bar, 20 µm; n = 3 mice/group. **c**, Immunofluorescence detection of lipid droplets in microglia using BODIPY and IBA1 co-immunostaining during the acute and chronic stage post-MCAO. Scale bar, 500 µm. The low panel shows a higher-magnification image for detailed visualization. Scale bar, 50 µm. Representative images from one of three independent experiments are shown. **d, e**, Filipin III (pink pseudo‐color) staining was employed to assess free cholesterol accumulation in microglia during the chronic phase post-MCAO. Scale bar, 20 µm; n = 3 mice/group. **f**, IBA1 immunostaining and polarized light microscopy (PLM) detecting needle-like cholesterol crystals (CCs) in the infarct area on day 30 and day 90 post-MCAO. Scale bar, 50 µm. Representative images from one of three independent experiments are shown. **g**, PLM shows birefringent color changes in CCs at days 3, 30, and 90 post-MCAO. Anisotropic crystalline nature confirmed by orientation-dependent interference colors using a first-order λ compensator. Scale bar, 20 µm. Representative images from one of three independent experiments are shown. **h**, Gene set enrichment analysis (GSEA) revealed changes in the cholesterol metabolism pathway across pairwise comparisons between days 3 vs 30, days 3 vs 90, and days 30 vs 90 post-MCAO. Normalized enrichment scores (NES) and *P* value for each gene set are displayed. n = 4 mice/group for sham and MCAO-30d groups; n = 3 mice/group for MCAO-3d and MCAO-90d groups. **i**, KEGG analysis reveals upregulation of cholesterol metabolism-related genes at days 3, 30, and 90 post-MCAO compared to sham controls. NES and *P* value for each gene set are displayed. n = 4 mice/group for sham and MCAO-30d groups; n = 3 mice/group for MCAO-3d and MCAO-90d groups. Data are presented as mean ± SD. Statistical significance was assessed by one-way ANOVA (**b, e**) followed by Dunnett’s multiple comparisons test, a one-sided hypergeometric test, and P-values were adjusted for multiple comparisons to generate q-values (**h**), or a one-sided hypergeometric test, and P-values were adjusted for multiple comparisons to generate q-values (**i**). Source data

Atherosclerosis is characterized by the formation of foam cells, primarily derived from monocytes or macrophages^18^. In a similar fashion, our study identified the persistent presence of foam cell-like structures by H&E staining in the lesion region after ischaemic injury (Extended Data Fig. 4a,b). This cellular hypertrophy, a hallmark of lipid-laden phagocytes in various pathological contexts, suggests substantial intracellular lipid accumulation, prompting further investigation into the specific lipid species and forms present in microglia. These observations prompted a systematic investigation into the specific forms of lipid and processing within microglia, including lipid droplets, cholesteryl esters, free cholesterol and cholesterol crystals.

**Fig. 3:**
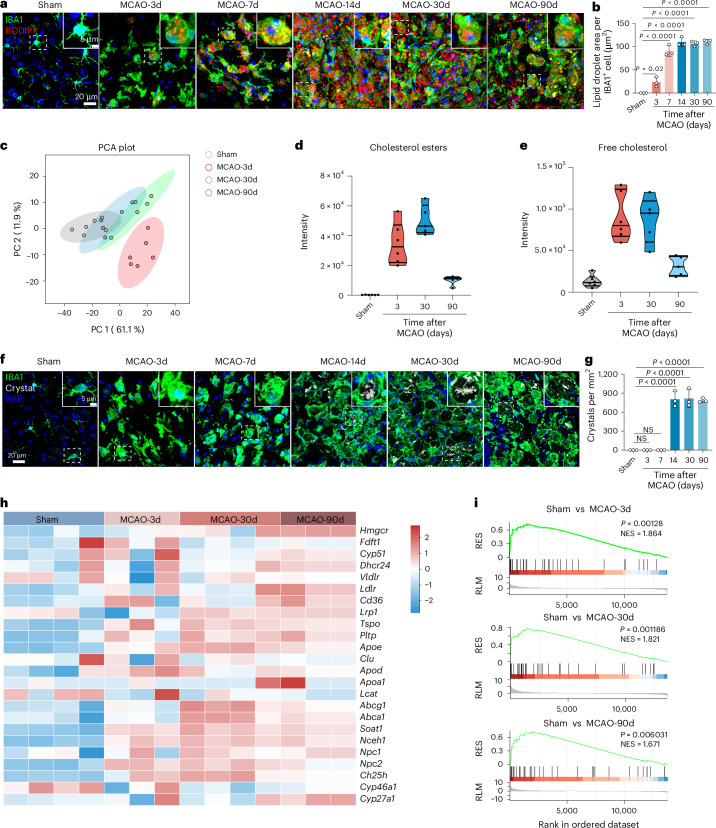
MCAO induces cholesterol metabolic reprogramming in microglia during the chronic stage. **a**, BODIPY and IBA1 double immunostaining on frozen sections showing lipid droplet accumulation in microglia during acute and chronic stages post MCAO. Lipid droplets are labelled with BODIPY; IBA1 marks microglia. Colocalization highlights microglia-specific lipid droplet accumulation. Top-right insets show higher magnification of the area inside white dashed rectangles. **b**, Quantitative data of the BODIPY^+^ area within IBA1⁺ cells across time points; *n* = 3 mice per group. **c**, Principal component analysis (PCA) of lipid species in microglia shows distinct clustering of sham and MCAO groups at days 3, 30 and 90, indicating time-dependent lipidomic shifts. **d**, Violin plots illustrating alterations in cholesterol esters within microglia, measured by a targeted lipidomics method by liquid chromatography–tandem mass spectrometry (LC–MS/MS) with multiple reaction monitoring during the acute and chronic stages post MCAO. Sham and MCAO-3d, *n* = 6 mice per group; MCAO-30d and MCAO-90d, *n* = 5 mice per group. **e**, Violin plots showing free cholesterol levels in microglia, measured by a targeted lipidomics method by LC–MS/MS with multiple reaction monitoring during the acute and chronic phases post MCAO. Sham and MCAO-3d, *n* = 6 mice per group; MCAO-30d and MCAO-90d, *n* = 5 mice per group. **f**, PLM and IBA1 immunostaining revealing cholesterol crystal (CC) deposition within microglia during acute and chronic phases; insets show higher magnification of the area inside white dashed rectangles. **g**, Quantification of crystal number per mm^2^ within IBA1^+^ cells; *n* = 3 mice per group. **h**, Heatmap analysis displaying changes in cholesterol metabolism-associated gene expression in microglia during the acute and chronic stage post MCAO. Sham and MCAO-3d, *n* = 4 mice per group; MCAO-30d and MCAO-90d, *n* = 3 mice per group. **i**, GSEA pathway enrichment analysis showing upregulation of cholesterol metabolism-associated pathways in microglia during the acute and chronic stage post MCAO. Sham and MCAO-3d, *n* = 4 mice per group; MCAO-30d and MCAO-90d, *n* = 3 mice per group. Data are mean and s.d. Statistical significance was assessed by one-way ANOVA followed by Dunnett’s multiple comparisons test (**b**,**g**) or a one-sided permutation test, and the resulting *P* values were adjusted for multiple comparisons (**i**). Source data

First, to identify the cell types accumulating neutral lipid stores, we performed co-staining with BODIPY 493/503 (a lipid droplet marker) and IBA1. This process revealed that lipid droplets were predominantly observed within microglia in the lesion area. Lipid droplet accumulation was minimal during the acute phase (days 3 and 7) but became markedly elevated during the chronic stage (days 14, 30 and 90) post MCAO (Fig. 3a,b and Extended Data Fig. 4c).

Next, we investigated the metabolism of cholesteryl esters, a major component stored within lipid droplets. Targeted lipidomic mass spectrometry analysis of sorted CD11b^+^ microglia showed alterations in cholesteryl ester profiles following MCAO (Fig. 3c,d). Consistent with changes in cholesteryl ester storage, bulk RNA-seq of these microglia revealed dynamic regulation of cholesteryl ester-metabolizing enzymes; *Soat1*, encoding the enzyme that synthesizes cholesteryl esters from free cholesterol, was significantly upregulated at days 3, 30 and 90 after MCAO, suggesting increased cholesteryl ester formation potential. Furthermore, *Nceh1*, encoding the enzyme for cholesteryl ester hydrolysis, was also significantly upregulated on days 30 and 90 (Fig. 3h). Together, these data point to active turnover and regulation of cholesteryl ester metabolism in microglia following stroke.

In addition to esterified cholesterol, we used Filipin III staining to identify and quantify free cholesterol^19,20^. The accumulation of free cholesterol was notably increased in microglia within lesion areas (Extended Data Fig. 4d,e), and lipidomics profiling further demonstrated a significant elevation in microglial free cholesterol levels during both the acute and chronic phases following MCAO (Fig. 3e).

As intracellular free cholesterol accumulation can surpass its solubility limit and precipitate into crystalline forms^21^, we next assessed cholesterol crystal formation. Using polarized light microscopy (PLM), a gold standard method for detecting birefringent crystalline structures^22–25^, we observed a significant increase in cholesterol crystals within the lesion area during the chronic stroke phase (days 14, 30 and 90) compared to sham controls and earlier time points (days 3 and 7) (Fig. 3f,g). Needle-like cholesterol crystals were observed in the lesion area by PLM at days 30 and 90 post MCAO (Extended Data Fig. 4f). Furthermore, these crystalline structures exhibited characteristic birefringence, displaying orientation-dependent interference colours under a λ-sub compensator, which confirmed their anisotropic crystalline nature (Extended Data Fig. 4g), highlighting cholesterol crystallization as a hallmark feature of chronic stroke pathology. Similar needle-like structures were also detected by transmission electron microscopy (Supplementary Fig. 1a,b).

Further transcriptomic analysis shed light on the cellular response to this altered cholesterol landscape. Transcriptomic analyses further revealed significant enrichment of cholesterol metabolism-related gene sets (based on GSEA) and upregulation of cholesterol efflux genes (*Abca1*, *Abcg1* and *Apoe*) during the chronic phase, while genes associated with de novo synthesis (*Hmgcr*, *Fdft1*, *Cyp51* and *Dhcr24*) showed minimal change (Fig. 3h,i). Notably, cholesterol uptake (*Ldlr*) and oxysterol synthesis (*Ch25h* and *Cyp27a1*) genes were markedly overexpressed at day 90 post stroke, highlighting dynamic shifts in cholesterol handling over time.

Parallel studies in female mice revealed similar patterns: BODIPY/IBA1 co-staining showed progressive lipid droplet accumulation in microglia across acute and chronic stages (3 and 90 days post MCAO) (Extended Data Fig. 2d,e), and PLM confirmed significant cholesterol crystal formation during the chronic phase (Extended Data Fig. 2f,g). Bulk RNA-seq revealed consistent upregulation of key cholesterol metabolism genes, including *Apoe*, *Tspo*, *Abcg1*, *Soat1*, *Npc2* and *Nceh1* during the acute and chronic stages after MCAO (Extended Data Fig. 2h).

**Extended Data Fig. 5:**
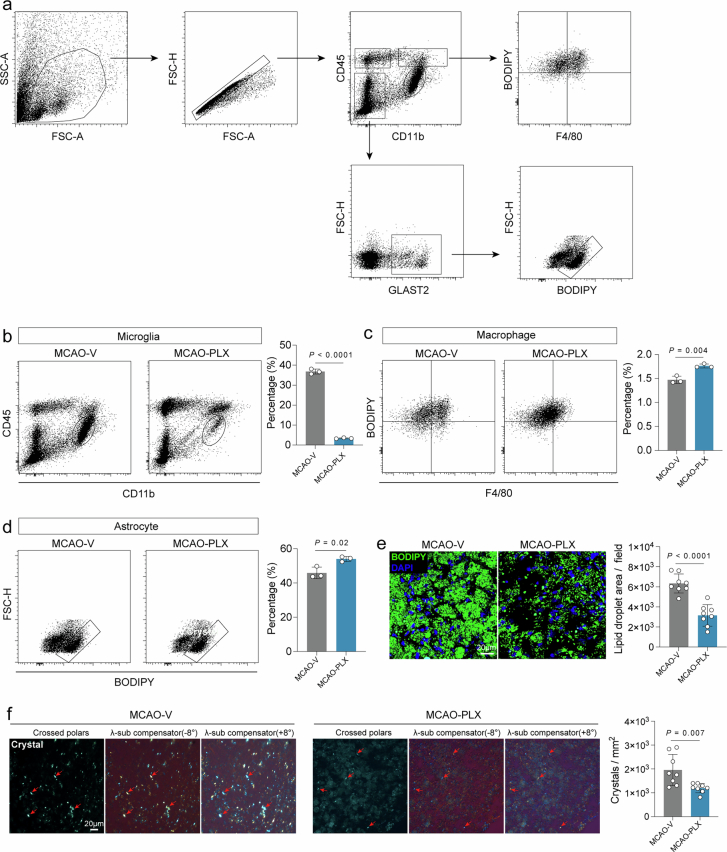
Effects of PLX5622 on cholesterol metabolism and brain cell populations post-MCAO. **a**, Flow cytometry gating strategy used to assess cellular lipid changes in response to PLX5622 (PLX) treatment. **b**, PLX treatment significantly depleted the microglial population following MCAO. n = 3 mice/group. **c**, Lipid droplet accumulation increased in brain macrophages after PLX treatment, suggesting a compensatory response. n = 3 mice/group. **d**, PLX treatment led to elevated lipid burden in astrocytes post-MCAO. n = 3 mice/group. **e**, BODIPY staining revealed reduced overall lipid deposition in the brain following PLX-induced microglial depletion. Scale bar, 20 µm; n = 8 mice/group. **f**, Polarized light microscopy (PLM) showed decreased cholesterol crystal (CC) accumulation in the brain after PLX treatment. The anisotropic crystalline nature was confirmed by orientation-dependent interference colors under PLM with a first-order λ compensator. Scale bar, 20 µm; n = 8 mice/group. Data are presented as mean ± SD. Statistical significance was assessed by a two-sided unpaired Student’s t-test (**b**, **c**, **d**, **e**, **f**). Source data

To further investigate the impact of microglial depletion on cholesterol homoeostasis following stroke, we administered PLX starting at 14 days post MCAO and continued treatment for 60 days. Flow cytometry was used to evaluate lipid droplet dynamics in astrocytes and peripheral macrophages, with the gating strategy outlined in Extended Data Fig. 5a. PLX effectively depleted microglia throughout the treatment period (Extended Data Fig. 5b). Notably, microglial depletion led to a significant increase in BODIPY^+^ signals in both peripheral macrophages and astrocytes (Extended Data Fig. 5c,d), indicating enhanced lipid redistribution to these cell populations. Consistently, BODIPY staining and PLM revealed a marked reduction in lipid droplet and cholesterol crystal accumulation in the brain following PLX treatment (Extended Data Fig. 5e,f).

**Extended Data Fig. 6:**
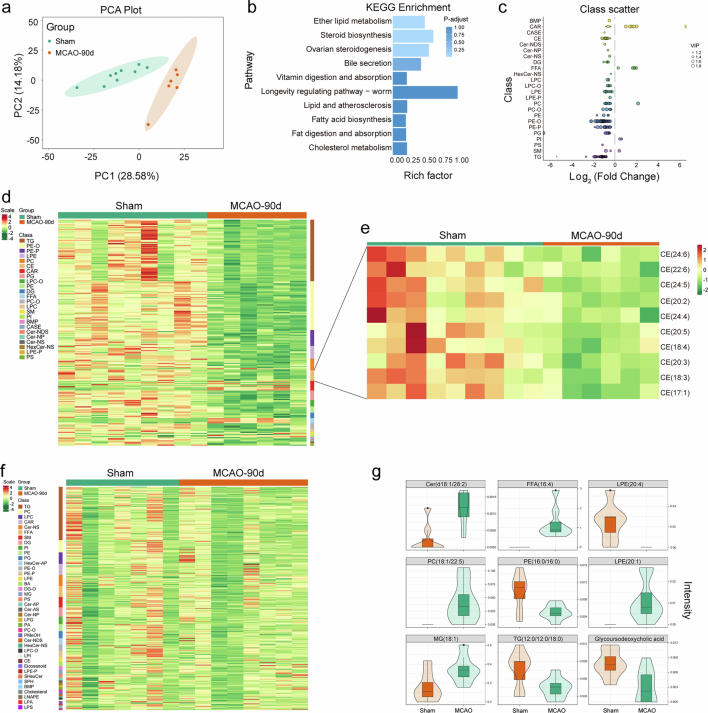
Changes in cholesterol and other lipid species in serum and CSF during the chronic stage post-MCAO. **a**, Principal coordinate analysis (PCA) of serum lipid profiles in sham-operated and 90-day post-MCAO mice reveals distinct clustering, indicating stroke-associated lipidomic alterations. **b**, Kyoto encyclopedia of genes and genomes (KEGG) pathway enrichment analysis identifies dysregulated lipid metabolism pathways at day 90 post-MCAO compared to the sham controls. n = 9 mice for the sham group; n = 6 mice for the MCAO-90d group. **c**, Class scatter analysis illustrates distinct lipid profile clustering between sham and MCAO-90d groups, indicating stroke-induced lipidomic changes. n = 9 mice for the sham group; n = 6 mice for the MCAO-90d group. **d**, Cluster analysis of serum lipidomics in sham-operated and MCAO-90d mice, showing differential lipid composition between groups. n = 9 mice for the sham group; n = 6 mice for the MCAO-90d group. **e**, Heatmap analysis depicting the abundance levels of cholesterol esters (CEs) in sham vs MCAO-90d groups, demonstrating significant changes in CEs composition. n = 9 mice for the sham group; n = 6 mice for the MCAO-90d group. **f**, Cluster analysis of cerebrospinal fluid (CSF) lipidomics in sham and MCAO-90d groups, highlighting stroke-induced lipidomic alterations in CSF. n = 7 mice for the sham group; n = 8 mice for the MCAO-90d group. **g**, Violin plots demonstrate a significant elevation of various lipid species in the MCAO-90d group compared to the sham group, indicating persistent dysregulation of lipid metabolism. The center line indicates the median, the box bounds represent the interquartile range (IQR) from the 25th to the 75th percentile, and the whiskers extend to the minimum and maximum values within 1.5 times the IQR. n = 7 mice for the sham group; n = 8 mice for the MCAO-90d group. Statistical significance was determined using a one-sided hypergeometric test, and P-values were adjusted for multiple comparisons to generate q-values(**b**).

To assess central and systemic lipidomic alterations in the chronic phase post MCAO, lipidomic profiling of serum and cerebrospinal fluid (CSF) was performed on day 90 post MCAO in male mice, focusing on cholesterol and related lipid species (Extended Data Fig. 6). Principal coordinate analysis visualized the distribution and clustering patterns of lipid species, revealing significant differences between the sham and MCAO-90d groups (Extended Data Fig. 6a). KEGG pathway enrichment analysis identified key alterations in lipid metabolism pathways at 90 days post MCAO, particularly in cholesterol metabolism and steroid biosynthesis (Extended Data Fig. 6b). Class scatter analysis further demonstrated distinct clustering of lipid profiles between the sham and MCAO-90d groups, underscoring the impact of ischaemic injury on lipid homoeostasis (Extended Data Fig. 6c). Heatmap analysis illustrated significant changes in the abundance levels of total lipids and cholesteryl esters, showing a clear separation between the sham and MCAO-90d groups (Extended Data Fig. 6d,e and Supplementary Table 3). Moreover, cluster analysis of CSF lipidomics confirmed distinct lipidomic signatures between the two groups, emphasizing the systemic effects of ischaemic injury on lipid composition (Extended Data Fig. 6f and Supplementary Table 4). Finally, violin plots demonstrated significant elevation of specific lipid species, including cholesteryl esters and phospholipids, in the MCAO-90d group compared to sham controls (Extended Data Fig. 6g). Collectively, these findings highlight the profound dysregulation of lipid metabolism following MCAO and provide critical insights into central and systemic lipidomic changes associated with ischaemic brain injury.

### MCAO induces SAM-foamy microglial clusters during the chronic stage in male mice

**Fig. 4:**
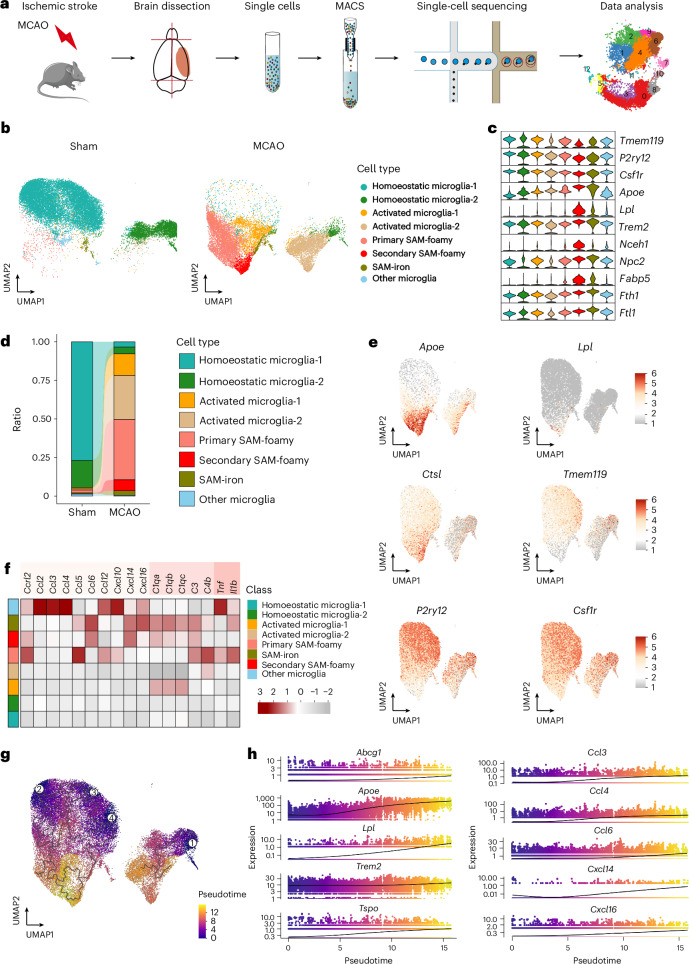
MCAO elicits SAM-foamy microglial clusters during the chronic stage. **a**, Workflow showing isolation and scRNA-seq of microglia from sham-operated and MCAO mice at 90 days post MCAO; *n* = 2 biological replicates per group. **b**, Uniform manifold approximation and projection (UMAP) of extracted microglia, coloured by inferred cluster identity from sham and MCAO male mice, showing distinct microglial subpopulations. SAM-iron, stroke-associated iron microglia. **c**, Violin plot illustrating gene signature expression across microglial clusters. **d**, Bar graphs showing cluster proportion, indicating MCAO-induced shifts in microglial composition. **e**, UMAP plots highlighting expression of representative genes associated with microglial activation, lipid metabolism and inflammation. **f**, Heatmap analysis depicting the expression of inflammation-related factors in microglia during the chronic stage post MCAO, revealing upregulated pro-inflammatory pathways. **g**, Trajectory reconstruction of all microglial cells, revealing four branches of microglial differentiation post MCAO, suggesting dynamic microglial transitions. **h**, Trajectory analysis highlighting the temporal expression patterns of key genes involved in cholesterol metabolism (for example, *Abcg1*, *Apoe*, *Lpl*, *Trem2* and *Tspo*) and inflammation-related factors (for example, *Ccl3*, *Ccl4*, *Ccl6*, *Cxcl14* and *Cxcl16*) across microglial differentiation states.

To gain deeper insights into microglial dynamics during the chronic phase post stroke, we performed scRNA-seq on CD11b^+^ cells isolated from the ipsilateral hemispheres of male mice 90 days after MCAO and from sham controls (Fig. 4a). Single-cell transcriptomes were obtained using the droplet-based 10× Genomics platform, and data processing, including quality control, principal component analysis and clustering, was conducted using the Seurat R package. Cell clusters were annotated based on lineage-defining genes and established immune cell markers.

**Extended Data Fig. 7:**
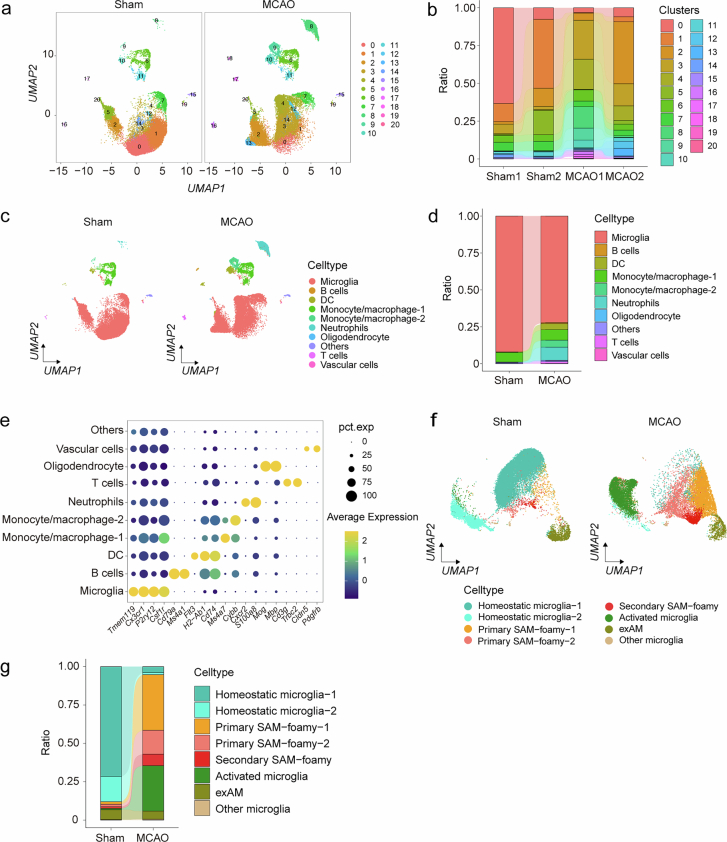
ScRNA-seq analysis of CD11b^+^ cells during the chronic stage post-MCAO. **a**, Uniform manifold approximation and projection (UMAP) of extracted CD11b⁺ cells from sham and MCAO mice, showing distinct cellular distributions between groups. **b**, Bar graphs depicting the proportion of each undefined cluster among CD11b⁺ cells, highlighting changes in cell populations following MCAO. **c**, UMAP colored by inferred cluster identity, illustrating transcriptional heterogeneity in CD11b⁺ cells from sham and MCAO mice. **d**, Bar graphs illustrating the distribution of defined immune and microglial clusters, revealing group-specific differences in subpopulation abundance. **e**, Dot plot displaying selected differentially expressed genes for each cluster. Dot size represents the percentage of expressing cells; Color represents average gene expression level, highlighting key transcriptomic alterations. **f**, Subclustering UMAP showing the distribution of microglial clusters in MCAO and sham groups, identifying distinct microglial subtypes. **g**, Bar graphs showing the proportion of each microglial cluster, demonstrating MCAO-induced shifts in microglial subpopulations.

Unsupervised clustering identified 21 distinct clusters (Extended Data Fig. 7a,b), which were further consolidated into ten major cell types based on significantly overrepresented genes in each cluster (Extended Data Fig. 7c–e). These included microglia (*Cx3cr1*, *P2ry12*, *Csf1r* and *Tmem119*), monocytes or macrophages (*Ms4a7* and *Cybb*), neutrophils (*Cxcr2* and *S100a8*), dendritic cells (*Flt3*, *H2-Ab1* and *Cd74*), T cells (*Cd3g* and *Trbc2*), B cells (*Cd79a* and *Ms4al*), oligodendrocytes (*Mbp* and *Mog*) and vascular cells (*Cldn5* and *Pdgfrb*) as well as an unidentified cluster labelled as ‘others’. A monocyte/macrophage-1 cluster, characterized by high *Pf4* and *Ms4a7* expression, probably represents resident macrophages.

Further analysis focused on microglial subsets, excluding a contaminating exAM cluster (associated with collagenase digestion) from sham and MCAO groups^26^ (Extended Data Fig. 7f,g). We identified eight distinct microglia clusters with high reproducibility (Fig. 4b,c). Among these, two clusters represented homoeostatic microglia, predominantly from sham mice, expressing signature genes such as *Tmem119*, *P2ry12* and *Csf1r*.

Two additional clusters were identified as SAM-foamy clusters, characterized by elevated expression of genes involved in lipid metabolism and phagocytosis, including *Apoe*, *Lpl*, *Trem2*, *Npc2*, *Nceh1* and *Fabp5* (Fig. 4c). Another distinct cluster represented stroke-associated iron microglia, showing lower expression of microglial signature genes but higher expression of iron-related genes such as *Fth1* and *Ftl1* (Fig. 4c). Uniform manifold approximation and projection highlights the spatial organization of microglia clusters and key genes expression patterns, including *Apoe*, *Lpl*, *Ctsl*, *Tmem119*, *P2ry12* and *Csf1r* (Fig. 4e).

Differential gene expression analysis revealed that the primary SAM-foamy cluster exhibited elevated expression of pro-inflammatory factors such as *Ccl5, Ccl10*, *Cxcl10*, *C3*, *C4b*, *Tnf* and *Il1b*, while the secondary SAM-foamy cluster showed increased expression of *Ccl6*, *Cxcl16*, *C1qa*, *C1qb*, *C1qc* and *C3* (Fig. 4f). To further investigate the temporal dynamics of microglial activation, we performed pseudotime trajectory analysis (Fig. 4g,h). This analysis enabled the reconstruction of gene expression trajectory, with a focus on five genes associated with cholesterol metabolism and inflammation. Notably, genes related to cholesterol metabolism, such as *Apoe*, *Lpl*, *Tspo* and *Abca1*, showed an increase before the upregulation of inflammatory factors, including *Ccl3*, *Ccl4*, *Ccl6*, *Cxcl14* and *Cxcl16* (Fig. 4h). This temporal relationship suggests a potential causal link between cholesterol accumulation and the pro-inflammatory phenotype observed in microglia during the chronic stage post stroke.

Our findings reveal the emergence of SAM-foamy clusters exhibiting a pronounced pro-inflammatory phenotype during the chronic stage post MCAO. Pseudotime analysis suggests that cholesterol accumulation may drive the pro-inflammatory property of microglia during ischaemic stroke recovery. These results provide insights into lipid-driven microglial activation and its role in chronic neuroinflammation following stroke.

### Cholesterol injection into the brain of male mice triggers sustained microglia activation

**Fig. 5:**
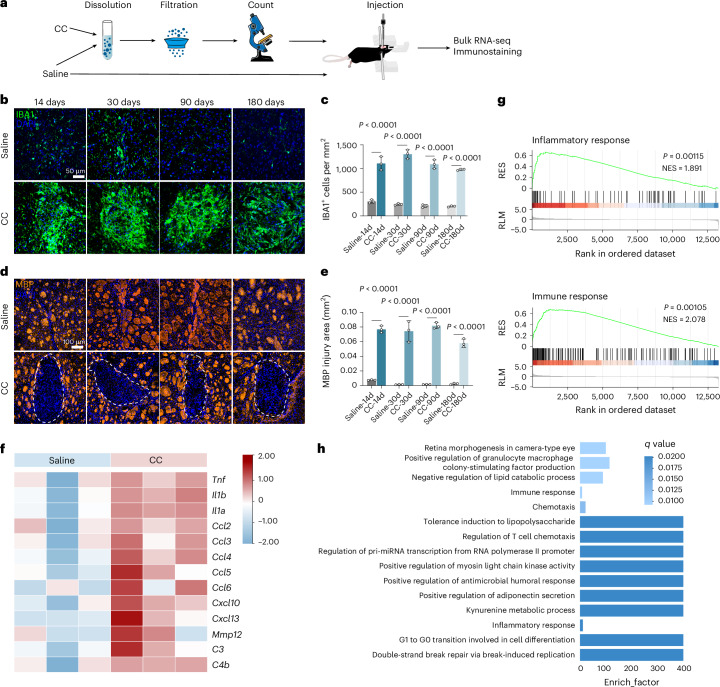
Cholesterol crystal deposition contributes to microglia-mediated chronic neuroinflammation and hinders white matter repair in mice. **a**, Experimental design for cholesterol crystal (CC) injection into the brain parenchyma, investigating their long-term effects on microglial activation and myelin repair. **b**,**c**, IBA1 immunostaining (**b**) and quantification (**c**) demonstrate sustained microglial activation after cholesterol crystal injection compared to saline controls; *n* = 3 mice per group. **d**,**e**, Cholesterol crystal injection immunostaining (**d**) and quantification (**e**) demonstrate impaired white matter repair, evidenced by increased MBP loss compared to vehicle controls; *n* = 3 mice per group. **f**, Heatmap showing upregulation expression of inflammation-related genes in microglia 90 days after cholesterol crystal injection compared to control; *n* = 3 mice per group. **g**, GSEA identifies upregulation of inflammatory and immune-related pathways in cholesterol crystal-injected microglia. NES and *P* values are shown for each gene set; *n* = 3 mice per group. **h**, Gene Ontology enrichment analysis revealed significant enrichment of immune and inflammatory response pathways, further supporting cholesterol crystal-induced neuroinflammation; *n* = 3 mice per group. Data are mean and s.d. Statistical significance was assessed by two-way ANOVA followed by Bonferroni’s multiple comparisons test (**c**,**e**) or a one-sided permutation test, and the resulting *P* values were adjusted for multiple comparisons (**g**). Source data

Building on our earlier findings, we reasoned that excessive cholesterol deposition, particularly cholesterol crystals, contributes to prolonged neuroinflammation following ischaemic stroke. To test this hypothesis, we directly injected cholesterol crystals into the brain parenchyma of rodents (Fig. 5a). Microglia exhibited sustained activation following cholesterol crystal injection, with a significantly increased microglial count in the cholesterol crystal-injected area compared to saline-injected controls from day 14 to 180 after injection (Fig. 5b,c). Cholesterol crystal injections caused persistent MBP lesions, indicating impaired white matter repair, compared to the control group (Fig. 5d,e).

**Extended Data Fig. 8:**
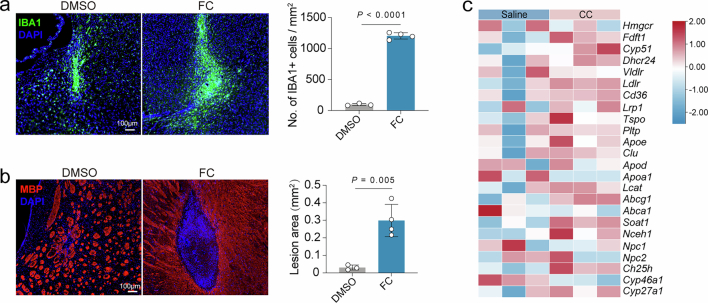
Free cholesterol overload induces microglial activation and myelin injury. **a, b**, Free cholesterol (FC) injection induced increased microglial activation (**a**) and myelin damage (**b**) during the chronic stage post-injection, compared to DMSO-treated controls. Scale bar, 100 µm; n = 3 mice for the DMSO group; n = 4 mice for the FC group. **c**, Changes in cholesterol metabolism-associated gene expression following cholesterol crystal (CC) injection, demonstrating molecular alterations in response to CC overload. n = 3 mice/group. Data are presented as mean ± SD. Statistical significance was assessed by a two-sided unpaired Student’s t-test (**a**, **b**). Source data

To investigate the molecular effects of cholesterol crystal deposition, we performed bulk RNA-seq of microglia isolated 90 days after cholesterol crystal injection. Differential gene expression analysis demonstrated elevated levels of inflammatory markers, including *Il1b*, *Il1a*, *Ccl4*, *Cxcl10* and *Cxcl13*, in microglia from the cholesterol crystal-injected brains compared to vehicle-treated controls (Fig. 5f). GSEA identified positively enriched gene sets related to inflammatory and immune responses (Fig. 5g). By comparison, Gene Ontology pathway analysis further indicated predominant enrichment of immune-related pathways in CD11b^+^ microglia from the cholesterol crystal-injected group (Fig. 5h). Analysis of cholesterol metabolism-related gene expression at 90 days after cholesterol crystal injection revealed overexpression of cholesterol metabolism-related genes compared to sham controls (Extended Data Fig. 8c).

To assess whether excessive free cholesterol contributes to neuroinflammation, we performed intracerebral injections of free cholesterol in healthy adult male mice. At 90 days after injection, free cholesterol injection induced sustained microglial activation, evidenced by increased microglial density at the injection site, along with persistent MBP loss, suggesting impaired white matter repair compared to dimethylsulfoxide (DMSO) controls (Extended Data Fig. 8a,b). We also administered BODIPY-cholesterol (BODIPY-C), a fluorescent cholesterol analogue, which confirmed the injection site through its fluorescence and similarly triggered prolonged microglial activation (Supplementary Fig. 2a,c).

To investigate whether microglial depletion mitigates cholesterol-induced brain injury, we administered PLX to normal male mice for 11 weeks to achieve sustained microglial depletion. Starting at week 3 of PLX treatment, BODIPY-C was injected into the brain. Histological analysis revealed that PLX treatment significantly reduced the lesion area in BODIPY-C-injected mice, as indicated by improved MBP staining compared to mice receiving BODIPY-C alone (Supplementary Fig. 2b)

These findings demonstrate that intracerebral injection of cholesterol into the brain parenchyma induces sustained microglial activation, which contributes to persistent neuroinflammation and impaired white matter repair.

### Genetic overexpression of *Cyp46a1* in microglia significantly ameliorates post-stroke brain lesions in male mice

**Fig. 6:**
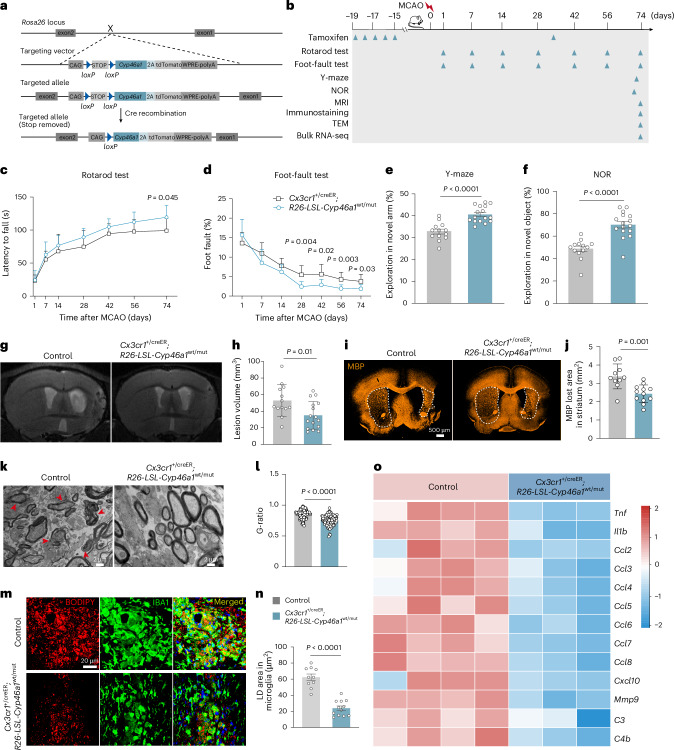
Genetic *Cyp46a1* overexpression in microglia significantly ameliorates neurological deficit post stroke. **a**, Schematic diagram illustrating the strategy used to generate microglia-specific *Cyp46a1* overexpression in adult male mice. **b**, Experimental timeline for testing the effects of microglial *Cyp46a1* overexpression during the chronic stage post MCAO. TEM, transmission electron microscopy. **c**,**d**, Motor and balance functions were assessed using the rotarod test (**c**) and foot-fault test (**d**) in MCAO mice with or without *Cyp46a1* overexpression. *Cyp46a1*-conditional knock-in (cKI), *n* = 16 mice; control, *n* = 13 mice. **e**,**f**, Cognitive function was evaluated using the Y-maze test (day 72 post MCAO) (**e**) and NOR test (day 70 post MCAO) (**f**). *Cyp46a1*-cKI, *n* = 16 mice; control, *n* = 13 mice. **g**,**h**, T2-weighted MRI (**g**) and quantification (**h**) showing reduced brain lesion volume in mice with *Cyp46a1* overexpression. *Cyp46a1*-cKI, *n* = 16 mice; control, *n* = 13 mice. **i**,**j**, MBP immunostaining (**i**) and quantification (**j**) indicate improved white matter integrity in *Cyp46a1*-overexpressing mice. *Cyp46a1*-cKI, *n* = 12 mice; control, *n* = 10 mice. **k**, Representative transmission electron microscopy images of the basal ganglia on day 74 post MCAO. Red arrowheads indicate demyelinated axons. **l**, The G-ratio (ratio of the inner axon diameter to the total outer fibre diameter) was compared between *Cyp46a1*-cKI and control mice. *Cyp46a1*-cKI, *n* = 105 axons from three mice; control, *n* = 98 axons from three mice. **m**,**n**, Lipid droplet (LD) visualization (**m**) and quantification (**n**) in microglia from the lesion area of *Cyp46a1*-cKI or control MCAO mice. *Cyp46a1*-cKI, *n* = 12 mice; control, *n* = 10 mice. **o**, Microglia-specific *Cyp46a1* overexpression suppressed pro-inflammatory gene expression in microglia post MCAO. *Cyp46a1*-cKI, *n* = 3 mice; control, *n* = 4 mice. Colour legend in **n** applies to all bar graphs. Data are presented mean and s.d. Statistical significance was assessed by two-way ANOVA (**c**,**d**) followed by Bonferroni’s multiple comparisons test or two-tailed unpaired Student’s *t*-test (**e**,**f**,**h**,**j**,**l**,**n**). Source data

**Extended Data Fig. 9:**
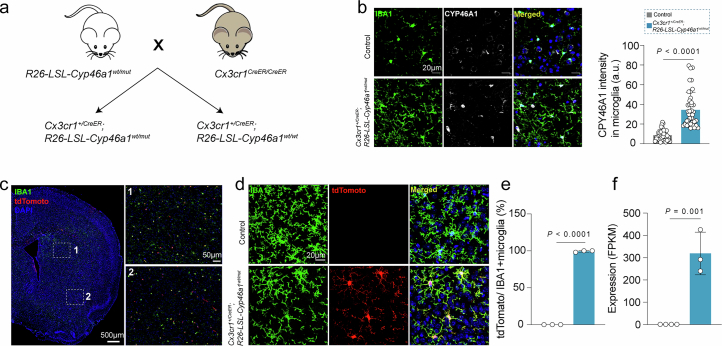
Reproductive strategies and verification of *Cyp46a1* transgenic mice. **a**, Breeding strategy for generating *Cyp46a1* overexpression of transgenic mice, illustrating the genetic approach used for microglia-specific overexpression. **b**, Double immunostaining for IBA1 and CYP46A1 confirms elevated *Cyp46a1* expression in microglia of *Cyp46a1*-cKI mice compared to controls. Scale bar, 20 µm. *Cyp46a1* cKI, n = 42 cells from three mice; control, n = 35 cells from three mice. **c-e**, Immunofluorescence analysis demonstrating microglia-specific expression of *Cyp46a1* in cKI mice, as shown by co-localization of IBA1⁺ (microglia marker) and tdTomato⁺ signals. Main image: scale bar, 500 µm; higher-magnification panel: scale bar, 50 µm (**c**). Scale bar, 20 µm(**d**); n = 3 mice/group. **f**, Bulk RNA-seq of CD11b⁺ cells sorted from control and *Cyp46a1*-cKI mice after MCAO reveals transcriptomic alterations associated with *Cyp46a1* overexpression. *Cyp46a1*-cKI, n = 3 mice; control, n = 4 mice. Data are presented as mean ± SD. Statistical significance was assessed by a two-sided unpaired Student’s t-test (**b**, **e**, **f**). Source data

CYP46A1, a brain-specific cholesterol 24-hydroxylase, is responsible for the majority of cholesterol clearance from the brain^27^. To investigate whether microglial-specific overexpression of *Cyp46a1* could enhance cholesterol clearance and improve outcome post stroke, we generated microglia-specific *Cyp46a1* knock-in mice (*Cx3cr1*^+/creER^; *R26*-*LSL-Cyp46a1*^wt/mut^) (Fig. 6a and Extended Data Fig. 9a). Tamoxifen was administered by intraperitoneal injection according to the following schedule: an initial course of one injection per day for five consecutive days, starting 19 days before MCAO surgery, followed by a single injection on postoperative day 35 (Fig. 6b). Successful induction of *Cyp46a1* overexpression in microglia was confirmed by immunostaining in *Cyp46a1* knock-in mice (Extended Data Fig. 9b–f).

To evaluate the effects of *Cyp46a1* overexpression on neurological and cognitive function, we conducted a series of behavioural tests after MCAO (Fig. 6b). Mice with microglia-specific overexpression of *Cyp46a1* (*Cyp46a1*-cKI) showed significantly longer rotarod latency and fewer foot faults on days 28, 42, 56 and 74 post MCAO, indicating improved motor coordination (Fig. 6c,d). *Cyp46a1*-cKI mice exhibited enhanced cognitive function, as evidenced by increased exploration of the novel arm in the Y-maze and greater interaction with the novel object in the recognition test (Fig. 6e,f).

A T2-weighted MRI on day 74 post MCAO showed significantly reduced lesion volumes in *Cyp46a1*-cKI mice compared to controls (Fig. 6g,h). Consistently, MBP staining revealed smaller lesion areas in the striatum of *Cyp46a1*-cKI mice (Fig. 6i,j). Electron microscopy further confirmed improved myelin integrity, as indicated by significantly better G-ratios in *Cyp46a1*-cKI mice during the chronic stage post MCAO (Fig. 6k,l).

Lipid droplet accumulation in microglia was significantly reduced in *Cyp46a1*-cKI mice compared to controls (Fig. 6m,n), suggesting that enhanced cholesterol metabolism alleviates lipid overload post stroke. Bulk RNA-seq of sorted microglial cells at day 74 post MCAO revealed a significant reduction of inflammatory factors, suggesting potential anti-inflammatory effects of *Cyp46a1* overexpression (Fig. 6o).

Together, these findings demonstrate that microglia-specific *Cyp46a1* overexpression reduces brain lesion volume, improves myelin integrity, enhances functional recovery, reduces lipid droplet accumulation in microglia and suppresses inflammation, highlighting its therapeutic potential in ischaemic stroke recovery.

### EFV profoundly enhances neurorestoration after ischaemic stroke in male mice

**Fig. 7:**
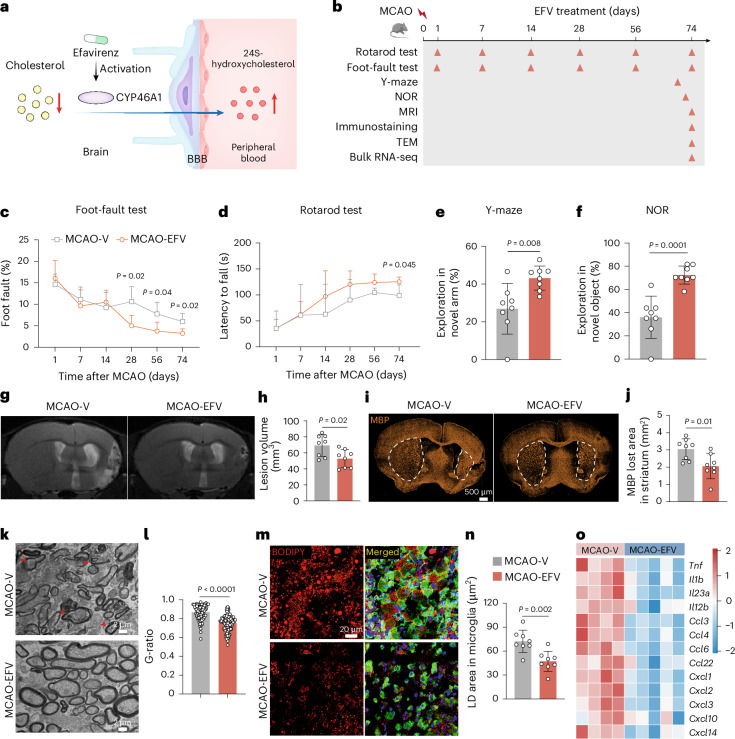
EFV, a CYP46A1 activator, enhances neurorestoration after stroke. **a**, Schematic illustrating the mechanism of EFV as a CYP46A1 activator that promotes cholesterol metabolism and neurorestoration post stroke. **b**, Experimental timeline depicting EFV or vehicle treatment in MCAO male mice during the chronic phase. **c**,**d**, Motor and balance functions were assessed using foot-fault (**c**) and rotarod (**d**) tests in MCAO mice treated with EFV or vehicle control; *n* = 8 mice per group. **e**,**f**, Cognitive function evaluation using the Y-maze test (day 72 post MCAO) (**e**) and NOR test (day 70 post MCAO) (**f**) in EFV and vehicle-treated MCAO mice; *n* = 8 mice per group. **g**,**h**, T2-weighted MRI images (**g**) and quantification (**h**) showing reduced brain lesion volume in EFV-treated mice compared to vehicle controls; *n* = 8 mice per group. **i**,**j**, MBP immunostaining (**i**) and quantification (**j**) showing improved white matter repair in EFV-treated mice compared to vehicle controls; *n* = 8 mice per group. **k**, Representative transmission electron microscope images of the basal ganglia at day 74 post MCAO; red arrowheads indicate demyelinated axons. **l**, G-ratio (ratio of the inner axon diameter to the total outer fibre diameter) was quantified and compared between EFV-treated and vehicle-treated groups; MCAO-EFV, *n* = 112 axons from three mice; MCAO-V, *n* = 78 axons from three mice. **m**,**n**, Lipid droplet accumulation (**m**) and quantification (**n**) in microglia from the lesion area was reduced by EFV treatment; *n* = 8 mice per group. **o**, Heatmap demonstrating downregulation of pro-inflammatory gene expression in microglia post MCAO following EFV treatment. MCAO-V, *n* = 4 mice; MCAO-EFV, *n* = 5 mice. Colour legend in **n** applies to all bar graphs. Data are mean and s.d. Statistical significance was assessed by two-way ANOVA (**c**,**d**) followed by Bonferroni’s multiple comparisons test or two-sided unpaired Student’s *t*-test (**e**,**f**,**h**,**j**,**l**,**n**). MCAO-V, MCAO-vehicle; MCAO-EFV, MCAO-efavirenz. Source data

Previous studies have demonstrated that the anti-HIV medication EFV activates CYP46A1 both in vitro and in vivo, increasing brain cholesterol turnover^13^ (Fig. 7a). In this study, EFV treatment was initiated 7 days post MCAO and administered daily until day 74 (Fig. 7b). Early neurological and cognitive functional outcomes were similar between EFV-treated and saline-treated MCAO mice (Fig. 7c–f). However, over time, EFV-treated MCAO mice exhibited significant neurological improvements, with reduced foot-fault rates on days 28, 56 and 74 post MCAO (Fig. 7c) and increased rotarod latency to fall on days 56 and 74 post MCAO (Fig. 7d). Cognitive performance was also significantly enhanced in EFV-treated mice compared to saline-treated controls, as measured by the Y-maze and novel object recognition tests (Fig. 7e,f).

T2-weighted MRI at 74 days after stroke showed a significant reduction in lesion volume in EFV-treated mice (Fig. 7g,h), while MBP staining revealed increased striatal myelin density compared to controls (Fig. 7i,j). Electron microscopy further confirmed enhanced myelin repair, with significantly improved G-ratios in EFV-treated mice (Fig. 7k,l). Additionally, EFV treatment markedly reduced lipid droplet accumulation in microglia (Fig. 7m,n). Bulk RNA-seq of sorted microglia at 74 days post MCAO revealed significantly downregulated pro-inflammatory transcripts following EFV treatment, indicating its potential anti-inflammatory effects (Fig. 7o).

Collectively, these findings indicate that EFV treatment initiated during the subacute phase of ischaemic stroke promotes neurorestoration by enhancing myelin repair, reducing microglial lipid accumulation and mitigating neuroinflammation, highlighting EFV as a promising therapeutic candidate for improving post-stroke recovery.

## Discussion

Our study demonstrates that ischaemic stroke induces prolonged microglial activation, characterized by cholesterol metabolic reprogramming and the formation of SAM-foamy clusters. These lipid-laden microglia exhibit sustained inflammation, contributing to chronic neuroinflammation and impaired white matter repair. Excessive cholesterol deposition not only prolongs microglial activation but also exacerbates secondary damage, highlighting its role as a key driver of persistent neuroinflammation.

Microglia rapidly activate in response to ischaemic injury, promoting leucocyte infiltration and exacerbating secondary damage. Although acute microglial activation is well characterized, the status and functional role of microglia during the chronic phase after stroke remains insufficiently explored. Translocator protein labelling suggests that microglial activation persists for weeks to months post stroke^5^. However, given that translocator protein is also expressed in astrocytes, its specificity for microglial activation is limited^28–30^. Immunofluorescence confirms prolonged microglial activation post MCAO^31^, yet morphology alone is insufficient to capture microglial dynamics. Activation involves proliferation, polarization and metabolic reprogramming, highlighting the need for a comprehensive assessment in addition to morphological changes.

To investigate the temporal dynamics of microglial responses and their inflammatory milieu, we analysed multiple time points from days 3 to 180 after stroke. The morphological assessment revealed spatially distinct microglial phenotypes: foamy and amoeboid microglia in the lesion core, indicative of phagocytic activity, and hypertrophied soma with retracted processes, indicative of sustained reactivity. These phenotypic states persisted long-term, suggesting spatial heterogeneity and prolonged microglial activation surpassing the acute phase. Bulk RNA-seq further demonstrated a transcriptional signature dominated by pro-inflammatory gene upregulation, indicating a persistent inflammatory state. This prolonged activation resembles microglial responses observed in neurodegenerative diseases such as Alzheimer’s disease and Huntington’s disease, in which chronic neuroinflammation exacerbates neuronal dysfunction^32–34^. Similarly, in traumatic brain injury, sustained microglial activation is linked to secondary damage and impaired recovery^33,34^. These findings suggest that understanding the mechanisms driving chronic microglial activation is essential for developing targeted interventions to reduce neuroinflammation and enhance long-term stroke outcomes.

Ischaemic stroke disrupts brain structures, releasing cellular debris that is phagocytosed by microglia, thereby triggering cholesterol metabolic reprogramming. We analysed microglial cholesterol metabolism post stroke, identifying cholesterol deposition (cholesteryl esters, cholesterol crystals and free cholesterol), increased uptake or transport and oxysterol formation. Imaging confirmed cholesterol crystal accumulation, foam cell formation and lipid droplet presence in microglia within the lesion core, mirroring previously published acute-phase findings^35^. Using mass spectrometry, we measured the content of free cholesterol and cholesteryl esters in microglia after stroke. In the chronic phase post stroke, microglia displayed significant free cholesterol accumulation and formed cholesterol crystal, indicative of cholesterol dysregulation. Notably, although a high level of free cholesterol is a prerequisite for crystallization, the actual amount of visible crystals reflects the portion of free cholesterol that has overwhelmed the cell’s buffering capacities (such as esterification into lipid droplets and efflux, which reduce the pool of free cholesterol available for crystallization) and has met the specific biophysical conditions (supersaturation, compartmentalization and time) necessary for nucleation and growth^36,37^. This may reflect both the high concentration threshold needed for crystallization and a dynamic intracellular cholesterol balance; the upregulation of genes for esterification (*Soat1*) and hydrolysis (*Nceh1*) suggests active processing that could modulate free cholesterol availability for crystallization, potentially by directing it towards storage pools. Moreover, bulk RNA-seq revealed upregulation of *Abca1*, *Abcg1*, *Apoe* (efflux) and *Ldlr* (uptake), with minimal biosynthesis changes. scRNA-seq identified SAM-foamy microglial clusters expressing genes like *Apoe*, *Trem2*, *Abca1*, *Tspo* and inflammatory markers, indicating a pro-inflammatory state that may impair white matter repair, highlighting a dual role for microglial cholesterol metabolism in chronic stroke recovery.

Building on this observation, our findings reveal a strong link between cholesterol dysregulation and chronic microglial activation post stroke. Pseudo-temporal scRNA-seq analysis showed inflammatory gene upregulation following cholesterol-related gene expression, suggesting cholesterol accumulation drives prolonged activation. To model this process, we injected free cholesterol or cholesterol crystals into mouse brains, inducing sustained microglial activation with increased inflammatory gene expression, morphological changes and elevated microglial count, mirroring atherosclerosis pathology^16,38^.

The precise mechanism underlying cholesterol-induced microglial inflammation remains unclear. In macrophages, cholesterol accumulation enhances TLR signalling and activates the NLRP3 inflammasome, driving inflammation^16,39^. However, post-stroke microglia showed minimal changes in NLRP3 and TLRs, except for increased *Tlr8* expression, which may contribute to prolonged inflammation given its role in cytokine responses^40^. Activated macrophages produce 25-hydroxycholesterol, a key pro-inflammatory molecule in atherogenesis^41^. We found significant upregulation of *Ch25h*, the enzyme responsible for 25-hydroxycholesterol synthesis, in post-stroke microglia, suggesting its role in cholesterol-induced inflammation.

Excessive cholesterol accumulation prolongs microglial activation post stroke, highlighting the need to reduce cholesterol overload to mitigate chronic neuroinflammation. In atherosclerosis, promoting cholesterol efflux in macrophages has shown therapeutic promise^42^. Inspired by this possibility, we aimed to enhance microglial cholesterol efflux to attenuate the inflammatory response. CYP46A1, a brain-specific cholesterol 24-hydroxylase^27^, facilitates cholesterol clearance by converting it into 24S-hydroxycholesterol, which exits the central nervous system by circulation^43^. Increasing *Cyp46a1* expression has shown benefits in Alzheimer’s disease and Niemann–Pick disease^27,44^. Here, microglia-specific *Cyp46a1* overexpression significantly improved stroke-induced neurological deficits, cognitive function and myelin repair, underscoring its neurorestorative potential. To enhance clinical applicability, we used EFV, an FDA-approved drug that crosses the BBB and allosterically activates CYP46A1 at low doses^45^. EFV boosted cholesterol efflux, reduced inflammation and replicated the neuroprotective effects of *Cyp46a1* overexpression, improving stroke recovery. These findings highlight CYP46A1 activation as a promising strategy for post-stroke therapy.

This study has several limitations. We focused on microglia, potentially overlooking the roles of astrocytes, neurons and immune cells. Single-cell or spatial transcriptomics could improve understanding. The pathway linking cholesterol deposition to inflammation remains unclear. MACS ensured viability but lacked purity, with CD11b⁺ populations including other immune cells; scRNA-seq could refine specificity. The study primarily used male mice to minimize variability from hormonal cycles, which limits the generalizability of the findings. Future studies should include both sexes to capture sex-specific stroke responses.

In conclusion, this study highlights cholesterol dysregulation in microglia as a key driver of chronic neuroinflammation and secondary injury following stroke. Therapeutic strategies targeting CYP46A1-mediated cholesterol metabolism hold significant potential for mitigating long-term neuroinflammation, promoting white matter repair and enhancing functional recovery.

## Methods

### Animals

Adult male and female C57BL/6J mice (8–10 weeks old) were obtained from Beijing Vital River Laboratory Animal Technology. *R26*-*LSL*-*Cyp46a1* mice were purchased from Shanghai Model Organisms Center, and *Cx3cr1*-*creER*-*eYFP* mice were sourced from The Jackson Laboratory. All procedures complied with National Institutes of Health guidelines for animal care and were approved by the Animal Research Ethics Committee, Capital Medical University (approval no. AEEI-2021-295). The mice were accommodated in specific-pathogen-free conditions with a 12 h–12 h light–dark cycle, maintaining an ambient temperature of 21–24 °C and 50% humidity, and with ad libitum access to water and food (SPF (Beijing) Biotechnology). Efforts were made to minimize animal use and ensure welfare by implementing measures to reduce pain and discomfort. All animal experiments were conducted in a randomized and blinded manner.

### MCAO

Transient focal ischaemic stroke was induced using the transient MCAO model^46,47^. Adult mice (8–10 weeks old) were anaesthetized with 1.5% isoflurane, and body temperature was maintained at 37 ± 0.5 °C. A midline neck incision was made to expose the left common carotid artery, which was ligated along with the external carotid artery. A microvascular clip temporarily blocked retrograde perfusion of the internal carotid artery. MCAO was achieved by inserting a silicon-coated 6-0 nylon filament (Doccol) into the common carotid artery. Cerebral blood flow was monitored by Laser Doppler (PeriFlux 5001, Perimed) to confirm occlusion. After 35 min, the filament was withdrawn for reperfusion, and the wound was closed. Sham-operated mice underwent the same procedure, except the filament was removed immediately without inducing ischaemia.

### Genotyping of mice

Genomic DNA was extracted from mouse toe clips collected during weaning. To genotype the conditional allele, two PCR reactions were performed. The wild-type *Rosa26* allele was identified with primers P1 (5′-TCAGATTCTTTTATAGGGGACACA-3′) and P2 (5′-TAAAGGCCACTCAATGCTCACTAA-3′), which generate a 967 bp product. The *LSL*-*Cyp46a1* knock-in allele was detected with primers P3 (5′-GAGCAGCTGGTGGAAATCCT-3′) and P4 (5′-ATCGTCCTGAGCTCCCTCTT-3′), yielding a 384 bp fragment. The wild-type *Cx3cr1* allele was amplified using primers *Cx3cr1*-creERP1(F) (5′-TGCCCCCTTCAGGACTCAACAAA-3′) and *Cx3cr1*-creERP2(R) (5′-AATATGCCCCCAAAGAAGCCAATG-3′), which produce an 867 bp band. The *Cx3cr1-creERT2* mutant allele was identified using primers *Cx3cr1*-creERP3(F) (5′-TGCATGCAGCCAGTGAGAACC-3′) and *Cx3cr1*-creERP4(R) (5′-GGATCCGCCGCATAACCAGTG-3′), which produce a 554 bp band.

### Cholesterol crystals, free cholesterol and BODIPY-C injection

Cholesterol crystals were freshly prepared and filtered using a 20 μm cell strainer to collect crystals (size of ≤20 μm)^48,49^. The crystals were counted using a hemocytometer and diluted to a final concentration of 5,000 ± 500 crystals per 100 μl of saline. Free cholesterol was prepared by dissolving cholesterol (cholest-5-en-3β-ol, C8667, Sigma) in DMSO at a concentration of 1 mg ml^−1^. Moreover, the cholesterol derivative BODIPY-C (24618, Cayman Chemical) was dissolved in DMSO to prepare a stock solution at a concentration of 1 mg ml^−1^.

For intracranial injections, mice were anaesthetized with 3.5% isoflurane and maintained with 1.5% isoflurane. A scalp incision was made, and a craniotomy was drilled. Cholesterol crystals, free cholesterol or BODIPY-C was injected using a micropipette and automated pump at 200 nl min^−1^ through a 10 μl Hamilton syringe into the striatum (AP, +0.5 mm; ML, +1.5 mm; DV, +3.2 mm). After the injection, the syringe was slowly withdrawn after 5 min to prevent backflow. Wounds were cleaned and sutured.

### Drug administrations

#### PLX treatment

To pharmacologically deplete brain microglia, adult mice were provided an AIN-76A diet containing PLX (1.2 g PLX per kilogram of diet, MedChemExpress, prepared by SYSE Bio) ad libitum^50^. Control mice were provided a standard AIN-76A diet (SYSE Bio) ad libitum^50^. Dietary administration of PLX began 14 days after MCAO and was maintained for either 21 days or 60 days to ensure effective microglia depletion.

#### EFV treatment

EFV (HY-10572, MedChemExpress) was dissolved in a solution of 10% DMSO and 90% saline. The solution was administered by oral gavage at a dosage of 0.09 mg kg^−1^ day^−1^ from day 7 to day 74 after MCAO.

#### Tamoxifen treatment

To induce CreER-dependent recombination in adult mice, tamoxifen (T5648, Sigma‐Aldrich) was dissolved in corn oil at a concentration of 20 mg ml^−1^. Mice received intraperitoneally tamoxifen daily at a dose of 100 mg kg^−1^ body weight. The treatment protocol involved two phases: an initial 5-day course starting 19 days before MCAO surgery, followed by a single application on postoperative day 35.

### Neurological function assessment

Neurological function was assessed in MCAO and sham mice using rotarod and foot-fault tests. Evaluations were performed before surgery and at multiple post-surgery time points by an investigator blinded to group assignments.

#### Rotarod test

Motor coordination was assessed using a rotarod apparatus (Harvard Apparatus, model 5200) with a rotating drum accelerating from 4 to 40 rpm^51^. Latency to fall was recorded with a 300 s cutoff. Trials ended if the mouse fell, clung to the drum or completed three consecutive passive rotations. Each mouse completed three trials at 15 min intervals, and the mean latency was used for analysis.

#### Foot-fault test

Locomotor function was evaluated using the modified foot-fault test^52^ to assess forelimb placement accuracy. The percentage of left paw missteps (footfalls) relative to the total number of steps (100 steps) was calculated to determine placement dysfunction.

### Cognitive function evaluation

Spatial memory and cognitive abilities were evaluated during the chronic phase post MCAO using a novel object recognition test and Y-maze test, as described previously^18,22^.

#### Novel object recognition test

Mice were habituated to a bin (50 × 50 × 50 cm) for 5 min the day before testing. During the trial phase (5 min), mice explored two identical objects. Exploration included sniffing, pawing or whisker probing within 1 cm but excluded sitting or climbing. During retention delay (4 h), mice were removed. During the test phase (5 min), mice re-entered the bin with one novel and one old object. Exploration time was recorded, and the discrimination index was calculated as time spent on the novel object / total exploration time.

#### Y-maze

The maze had three arms, with one (novel arm) blocked during training. During training (5 min), mice explored two arms (start and familiar). During retention (4 h), the mice were removed. During testing (5 min), the mice re-entered with access to all three arms, including the novel arm. Entries into the novel arm were recorded as a percentage of total entries to assess memory.

### MRI measurements and analysis

The lesion volume in MCAO mice was quantified using a 7.0 Tesla small animal MRI scanner (Biospec 70/20 USR, Bruker BioSpin) equipped with a 72 mm volume transmitter coil and a dedicated mouse brain surface receiver coil. Mice were anaesthetized with 3.5% isoflurane (induction) and maintained at 1.5% isoflurane in a medical gas mixture (70% N_2_O, 30% O_2_). Physiological monitoring included respiratory rate measurement using a pillow sensor (SA Instruments) and maintenance of core body temperature at 37.0 ± 0.5 °C using a feedback-regulated heated water circulation system (Harvard Apparatus). T2-weighted axial brain images were acquired using a fat-suppressed RARE sequence (repetition time, 4,000 ms; echo time, 60 ms; matrix, 192 × 192; field of view, 19.2 × 19.2 mm). T2 mapping was performed using a Multislice Gradient Echo sequence. MRI data were analysed with ITK-SNAP (v.4.2.2; http://www.itksnap.org), defining infarct volume as hyperintense regions on T2-weighted images across the entire brain. To account for brain atrophy, the final lesion volume was calculated as lesion volume = contralateral parenchyma volume − (ipsilateral parenchyma volume − infarct volume).

### CSF and serum collection

CSF samples were collected for subsequent lipidomic analysis^53^. Adult C57BL/6J mice were anaesthetized with isoflurane and positioned in a stereotactic frame. A sagittal incision was made below the skull, and the neck muscles were separated to expose the cisterna magna. CSF was withdrawn using a glass capillary, centrifuged at 2,000*g*, 4 °C for 30 min to remove debris, and the supernatant was stored at −80 °C for analysis. The serum was collected from anaesthetized mice by terminal cardiac puncture, allowing the blood to clot before centrifugation at 2,000*g* for 30 min to isolate serum, followed by storage at −80 °C for targeted lipidomics analysis.

### Tissue preparation for pathology and immunohistochemistry

Adult mice were killed on days 3, 7, 14, 30, 90 and 180 post MCAO. Brain samples were collected and fixed overnight in 4% paraformaldehyde at 4 °C, followed by sequential dehydration in 15% and 30% sucrose solutions prepared in 1× PBS. The tissues were then embedded in optimal cutting temperature compound (Tissue-Tek), rapidly frozen in liquid nitrogen and sectioned into 20 μm coronal slices using a Leica CM1950 cryostat for immunohistochemistry and cholesterol visualization assessment, including Filipin III staining, cholesterol crystal visualization (by PLM) and BODIPY 493/503 staining.

Post-mortem injury-region brain tissue was obtained from W. Jin. Paraffin blocks were sectioned at 6 µm and mounted on charged slides. Sections were deparaffinized in xylene and rehydrated through graded ethanol.

### Immunohistochemistry

Paraffin human sections were subjected to antigen retrieval in 10 mM sodium citrate buffer (pH 6.0) at 95 °C for 5 min. Frozen brain sections were rinsed three times in 1× PBS for 10 min each. Sections were incubated in blocking buffer at room temperature (20–25 ℃) for 1 h. Sections were incubated overnight at 4 °C with primary antibodies diluted in blocking buffer. After PBS rinses, sections were incubated with secondary antibodies at room temperature for 1 h, followed by three PBS washes. Sections were mounted with DAPI-containing anti-fade medium (Abcam, ab104139). Primary antibodies included IBA1 (1:1,000; Wako, 01919741), CYP46A1 (1:200; Proteintech, 12486-1-AP) and MBP (1:500; Abcam, ab7349). For each frozen sample, three slides were analysed, each containing three fields of view. Images were digitized and analysed using ZEN 2.1 (Carl Zeiss) or ImageJ. IBA1⁺ microglial cell numbers were quantified automatically by threshold-based particle counting, expressed as cells per mm^2^.

### Measurement of myelin loss

Myelin loss after MCAO was assessed volumetrically using MBP-stained coronal sections. Every tenth section from bregma 0 mm to +1 mm was prepared, resulting in three sections per sample. Whole-brain MBP fluorescence images were acquired using the Mica microscope. Myelin loss was quantified by analysing the MBP^+^ area within the striatum. The myelin loss area was calculated as follows: MBP lost area = area of contralateral striatum − area of ipsilateral striatum.

### H&E staining

H&E staining (G1120, Solarbio) was used to assess foam cells in lesion regions during the chronic phase post MCAO. Frozen brain sections were stained with haematoxylin for 10 min, rinsed with tap water, differentiated for 2 min and counterstained with eosin Y for 20 s. Sections were then dehydrated through graded ethanol, cleared in xylene and mounted with resin. For each sample, three sections were prepared, and three representative fields per section were imaged for analysis.

### Cholesterol visualization assessment

Free cholesterol was visualized using Filipin III (refs. ^19,20,54^) (B6034, APExBIO). Frozen brain sections were washed using 1× PBS and incubated in the dark with Filipin III solution (0.5 mg ml^−1^) for 2 h at room temperature. Images were captured using a Leica SP8 lightning microscope with 340–380 nm excitation. ImageJ (v.1.54g; National Institutes of Health) was used to differentiate Filipin III-positive signals from autofluorescence. Results are expressed as the Filipin III-positive area per IBA1^+^ microglia.

Cholesterol crystal in cryosectioned brain tissue were identified by their characteristic birefringence using PLM (Zeiss LSM900 or Zeiss Imager M2)^55^. The critical interference colour changes are observed when a rotatable λ-sub compensator plate (Compensator Lambda 6 × 20 mm; rotary, ±8°; Zeiss, 453710) is inserted into the light path^56–58^. The number of crystals was measured using ImageJ. The ‘threshold’ tool was applied to isolate birefringent signals based on brightness and morphology. The ‘analyse particles’ function was used to count crystals. Results are expressed as the number of crystals per mm^2^, with three slides analysed per sample and three fields of view quantified per slide.

Lipid droplet staining in frozen brain sections was performed with BODIPY 493/503 (BODIPY, HY-W090090, MedChemExpress)^54^. Image analysis was performed using ImageJ. The threshold was adjusted to isolate lipid droplet signals from the background. Results are expressed as the total lipid droplet area per microglia, with three slides analysed per sample and three fields of view quantified per slide.

### Transmission electron microscopy

Transmission electron microscopy was performed. Mice were perfused with cold phosphate buffer, followed by 4% paraformaldehyde and 2.5% glutaraldehyde. The lesion region was micro-dissected into 1 mm^3^ blocks and fixed in 2% glutaraldehyde for 24 h. Samples were washed, post-fixed in 1% osmium tetroxide and 1% potassium ferricyanide for 1 h, dehydrated through graded ethanol (30–100%), treated with 100% propylene oxide and infiltrated overnight with a 1:1 propylene oxide mixture at room temperature. The tissue was embedded, cured at 37 °C overnight and hardened at 65 °C for 2 days. Ultra-thin 60 nm sections were prepared using a Leica UCT ultramicrotome with a diamond knife (Diatome), then stained with uranyl acetate and lead citrate. Images were acquired using a JEOL JEM 2100 transmission electron microscope (100 kV) fitted with an AMT digital camera. Three sections from each animal were analysed. Nine images were captured from random lesion regions within the basal ganglia. At ×5,000 magnification, the axonal and whole-fibre circumferences of at least 30 randomly selected axons per animal were traced blindly. Axonal and total fibre circumferences were traced using ImageJ, and G-ratios were calculated as follows: G-ratio = inner axonal diameter/outer diameter (axonal diameter + total myelin sheath thickness).

### Analysis of morphometric features

Microglial morphological analysis was performed using Imaris (v.9.2.1) software (Bitplane Imaris)^59,60^. Three-dimensional reconstruction of microglial processes was performed using the FilamentTracer plugin with a semi-automated algorithm, which minimized manual selection bias by integrating adaptive seeding point detection and branch connectivity analysis. The tracing parameters were configured with a maximum seeding point diameter of 12 µm and a spatial resolution of 1 µm. Disconnected segments were removed by applying a smoothness threshold of 0.6 µm to ensure structural continuity and minimize artefacts. To ensure data quality, microglial cells located at image borders or exhibiting incomplete tracing were manually excluded from subsequent analysis. The reconstructed skeletons were exported in the native .ims format (Imaris-specific hierarchical data structure) and subsequently converted to the standardized .swc format to ensure cross-platform compatibility and facilitate downstream analysis. Three-dimensional spatial coordinates (*x*, *y*, *z*) and process diameters were extracted using the ImarisReader toolbox (MATLAB R202X), enabling precise quantification of microglial morphology for downstream analysis. The morphological data underwent standardization using the NL Morphology Converter (NeuroLand), which automatically filtered out structural artefacts that were inconsistent with the .swc format specifications.

### Cell isolation and sorting

Microglia were isolated using MACS. Mice were anaesthetized with deep ketamine and xylazine and then killed, followed by perfusion with cold 1× PBS. The brain tissue (excluding the forebrain and cerebellum) was minced and digested with collagenase D (1 mg ml^−1^, Roche Diagnostics) at 37 °C for 45 min. The suspension was filtered through a 70 µm strainer (BD Falcon) and centrifuged on a 30% Percoll gradient (Cytiva, 17089109) to remove myelin. The cell pellet was washed and resuspended in cold 1× PBS with 0.5% BSA (Sigma-Aldrich, V900933). For microglia enrichment, CD11b MicroBeads (Miltenyi Biotec, 30093634) were incubated for 15 min, and CD11b-FITC (BioLegend, 101206) was added for 5 min at 4 °C to assess purity. The suspension was passed through an MS column (Miltenyi Biotec, 130042201), with a second passage for higher purity. Enriched CD11b⁺ cells were used for lipid mass spectrometry, scRNA-seq and bulk RNA-seq.

### Flow cytometry

Adult mice were killed, perfused with 1× PBS and the brain tissue was minced and digested with 1 mg ml^−1^ collagenase D at 37 °C for 45 min, with gentle agitation every 10 min. The digested tissue was filtered (100 μm strainer) and centrifuged on a 30% Percoll gradient (700*g*, 10 min) to remove myelin. The cell pellet was washed and resuspended in cold 1× PBS plus 0.5% BSA. Single-cell suspensions were Fc-blocked and incubated with surface antigen antibodies (30 min, on ice, in the dark). For lipid droplet staining, cells were incubated with 2 μM BODIPY 493/503 for 15 min at 37 °C. Flow cytometry was performed on a BD Aria III (BD Biosciences), and data were analysed using BD FACSDiva software. Antibodies used included CD45 (30-F11), CD11b (M1/70), F4/80 (BM8) and GLAST2 (IH3-18A3).

### scRNA-seq

CD11b^+^ cells were isolated from the ipsilateral brain of MCAO mice using MACS, and two mice were pooled per group. Viability (>80%) was assessed using the Cell Viability Counter System (Countstar). The single-cell suspension was diluted to 1,000 cells ml^−1^, loaded onto a 10× Genomics chip and processed on a Chromium Controller to generate gel-bead emulsions. Single-cell libraries were generated using the 3′-labelling kit (10× Genomics), library quality was determined by the Agilent High Sensitivity DNA Bioanalyzer chip and sequencing was performed on an Illumina NovaSeq X plus sequencer. scRNA-seq raw data were processed using Cell Ranger (v.7.0.0) (10× Genomics) and aligned to the GRCm38 (mm10) mouse reference genome. Downstream analysis, including uniform manifold approximation and projection, heatmap, graphs and violin plots, was performed using Seurat (v.3.1.1). Pseudotime analysis was conducted with Monocle (v.3).

### Bulk RNA-seq

Microglial RNA was extracted using the TRIzol reagent (15596026CN, Thermo Fisher). A total of 1 μg RNA per sample was prepared for bulk RNA-seq. Libraries were generated using the Hieff NGS Ultima Dual-mode mRNA Library Prep Kit for Illumina (Yeasen Biotechnology). mRNA was purified using poly-T oligo-attached magnetic beads, and library fragments were purified with the AMPure XP system (Beckman Coulter). PCR amplification was performed using Phusion high-fidelity DNA polymerase, Universal PCR primers and Index (X) Primer. The libraries were sequenced on an Illumina NovaSeq platform to generate 150 bp paired-end reads. High-quality sequencing reads were aligned to the reference genome using Hisat2. Identification of differentially expressed genes was performed using DESeq2, applying significance criteria of *P* < 0.05 and fold change of ≥2. Gene Ontology enrichment analysis for the identified differentially expressed genes was conducted using the clusterProfiler package. KEGG pathway enrichment analysis was carried out using the KOBAS database in conjunction with the clusterProfiler software to identify statistically enriched biological pathways.

### Lipid extraction and lipidomics

#### Sample preparation and lipid extraction

Frozen samples were thawed on ice and vortexed for 10 s. Lipid extraction was performed directly without saponification. Each sample was mixed with 1 ml of the extraction solvent (methyl tert-butyl ether/methanol, 3:1, v/v) containing an internal standard mixture (Supplementary Table 1). After vortexing for 15 min, 100 μl of ultrapure water was added. The mixture was vortexed for 1 min and centrifuged at 14,000*g* for 10 min. The upper organic phase was collected, dried under vacuum and reconstituted in 200 μl of ACN/IPA (1:1, v/v) for liquid chromatography–tandem mass spectrometry analysis.

#### Lipid identification

The qualitative experiment was performed on an Ultimate 3000 UHPLC system coupled to a Q-Orbitrap mass spectrometer (Q Exactive, Thermo Scientific). The instrument was calibrated before measurements were taken to minimize detection error in the accurate mass of lipids. The calibration solution of positive and negative ion modes was purchased from Thermo Scientific. The lipids were assigned based on accurate mass value (<10 ppm) and isotopic patterns, and tandem mass spectrometry spectra were compared with HMDB, METLIN and LIPID MAPS databases.

Lipid separation was conducted using a Thermo Accucore C30 column (2.6 μm, 2.1 mm × 100 mm). The solvent system comprised mobile phase A (acetonitrile/water, 60:40, v/v) with 0.1% formic acid and 10 mM ammonium formate and mobile phase B (acetonitrile/isopropanol, 10:90, v/v) with 0.1% formic acid and 10 mM ammonium formate. The gradient conditions were as follows: 0–2 min, 20–30% B; 2–4 min, 30–60% B; 4–9 min, 60–85% B; 9–14 min, 85–90% B; 14–15.5 min, 90–95% B; 15.5–17.3 min, 95–95% B; 17.30–17.31 min, 95–20% B; and 17.31–20 min, 20–20% B. The flow rate was 0.35 ml min^−1^, the column temperature was 45 °C, and the injection volume was 2 μl.

The mass spectrometer parameters were optimized as follows: sheath gas, 45 arb.; auxiliary gas, 10 arb.; auxiliary gas heater temperature, 220 °C; spray voltage, 3.5 kV or −3.2 kV; capillary temperature, 350 °C. For a full scan, the automatic gain control target and maximum injection time were 3 × 10^6^ and 200 ms, respectively, with a resolution of 70,000. For parallel reaction monitoring, the resolution was set at 17,500, and the automatic gain control target and maximum injection time were 2 × 10^5^ and 100 ms, respectively. The normalized collision energy was set as 15%, 30% and 45%. The isolation window was set at 1 Da.

#### Targeted lipidomics analysis

The relative quantification of lipids was performed using a targeted lipidomics method via liquid chromatography–tandem mass spectrometry with multiple reaction monitoring. The analysis was carried out using an ExionLC UHPLC system coupled with a QTRAP 6500+ Mass Spectrometer (AB SCIEX), controlled by Analyst (v.1.6.3) software (AB SCIEX).

Lipid separation was carried out using the previously described C30 column and gradient elution protocol. Lipids were detected using the QTRAP 6500+ mass spectrometer equipped with a Turbo Ion-Spray source, operating in both positive and negative ion modes. The source parameters were as follows: temperature, 500 °C; ion-spray voltage, +5.5 kV (positive mode) or −4.5 kV (negative mode); curtain gas, 35 psi; GS1, 45 psi; GS2, 55 psi. Instrument tuning and mass calibration were performed using polypropylene glycol at concentrations of 10 μmol l^−1^ and 100 μmol l^−1^ in QQQ and LIT modes, respectively. Multiple reaction monitoring transitions were individually optimized for each lipid species, with nitrogen as the collision gas. Each multiple reaction monitoring transition was scheduled based on lipid elution profiles.

### Statistical analysis

All measurements were performed by investigators blinded to each group’s allocation and experimental conditions. No animals or data points were excluded from the analyses. No statistical methods were used to pre-determine sample sizes, but our sample sizes are similar to those reported in previous publications^59^. Data distribution was assumed to be normal, although this was not formally tested^61^. Statistical analyses were conducted using GraphPad Prism (v.10.2.3, GraphPad). For two-group comparisons, unpaired two-tailed Student’s *t*-tests were used. For comparisons involving three or more groups, one-way ANOVA followed by Dunnett’s multiple comparisons test or Bonferroni’s multiple comparisons test was applied, as appropriate. For experiments involving two independent variables, two-way ANOVA with Bonferroni’s multiple comparisons test was used. The variable *n* denotes the number of biological replicates. Unless stated otherwise, data are presented as mean ± s.d., and *P* < 0.05 was considered statistically significant. Specific statistical tests are indicated in the corresponding figure legends.

### Reporting summary

Further information on research design is available in the Nature Portfolio Reporting Summary linked to this article.

## Supplementary information

Supplementary Information Materials & Methods, Abbreviations, Supplementary Figs. 1–7, Supplementary Tables 1–4

Reporting Summary

Supplementary Table 1 Medical Animal Target Lipidomics: Internal Standard Information

Supplementary Table 2 Changes in lipid species in CD11^+^ microglia during the acute and chronic stage post-MCAO

Supplementary Table 3 Changes in lipid species in serum during the chronic stage post-MCAO

Supplementary Table 4 Changes in lipid species in cerebrospinal fluid (CSF) during the chronic stage post-MCAO

## Source data

Source Data Fig. 1 Statistical Source Data

Source Data Fig. 2 Statistical Source Data

Source Data Fig. 3 Statistical Source Data

Source Data Fig. 5 Statistical Source Data

Source Data Fig. 6 Statistical Source Data

Source Data Fig. 7 Statistical Source Data

Source Data Extended Data Fig./Table 1 Statistical Source Data

Source Data Extended Data Fig./Table 2 Statistical Source Data

Source Data Extended Data Fig./Table 3 Statistical Source Data

Source Data Extended Data Fig./Table 4 Statistical Source Data

Source Data Extended Data Fig./Table 5 Statistical Source Data

Source Data Extended Data Fig./Table 8 Statistical Source Data

Source Data Extended Data Fig./Table 9 Statistical Source Data

## Data Availability

The raw sequencing data for scRNA-seq and bulk RNA-seq reported in this paper have been deposited in the Genome Sequence Archive and are publicly available under accession codes CRA017588 and CRA017573. The single-cell transcriptomic matrix was submitted to OMIX, China National Center for Bioinformation/Beijing Institute of Genomics, Chinese Academy of Sciences (https://ngdc.cncb.ac.cn/omix; accession no. OMIX011437). All other data supporting the findings of this study, including supplementary figures and tables, are included in the paper or its supplementary materials. Source data are provided with this paper.

## Code availability

No new code was generated in this study.
