# Supplementary material for: Cholesterol metabolic reprogramming mediates microglia-induced chronic neuroinflammation and hinders neurorestoration following stroke
Source: Nat Metab. 2025 Sep 23;7(10):2099–116. doi: 10.1038/s42255-025-01379-7 (PMC12552130; doi:10.1038/s42255-025-01379-7)
Supplement: Supplementary file 1 — Materials & Methods, Abbreviations, Supplementary Figs. 1–7, Supplementary Tables 1–4 [file 42255_2025_1379_MOESM1_ESM.pdf]

nature metabolism

Supplementary information https://doi.org/10.1038/s42255-025-01379-7

Cholesterol metabolic reprogramming mediates microglia-induced chronic neuroinflammation and hinders neurorestoration following stroke

In the format provided by the authors and unedited

Supplementary Information

Abbreviations

CC, cholesterol crystals; FC, free cholesterol; LD, lipid droplet; 24S-OHC, 24S-hydroxycholesterol; 25-OHC, 25-hydroxycholesterol; 27-OHC, 27-hydroxycholesterol; SAM-foamy, stroke-associated foamy microglia; CYP46A1, cytochrome P450 family 46 subfamily A member 1; EFV, Efavirenz; PLM, polarized light microscopy; MBP, myelin basic protein; IBA1, ionized calcium-binding adaptor molecule 1; H&E, Hematoxylin-eosin; TEM, transmission electron microscopy; TSPO, translocator protein; CSF, cerebrospinal fluid; MCAO, middle cerebral artery occlusion; PLX, PLX5622; TLR, Toll-like receptor; RES, running enrichment score; RLM, ranked list metric; NES, normalized enrichment scores; GO, gene ontology; KEGG, kyoto encyclopedia of genes and genomes; UMAP, uniform manifold approximation and projection; PCA, principal component analysis; NOR, novel object recognition; GSEA, gene set enrichment analysis; scRNA-seq, single-cell RNA sequencing; bulk RNA-seq, bulk RNA sequencing; CNS, central nervous system; BBB, blood-brain barrier; AD, Alzheimer's disease; PD, Huntington's disease; BBB, blood-brain barrier; MACS, magnetic-activated cell sorting; MRI, magnetic resonance imaging; CAR, Acylcarnitine; CE, Cholesteryl ester; SPH, Sphingosine; DG, Diacylglycerol; MG, Monoacylglycerol; LPC, Lysophophatidylcholine; LPC-O, alkyl-Lysophophatidylcholine; LPE, Lysophosphatidylethanolamine; LPE-P, alkenyl-Lysophosphatidylethanolamine; LPG, Lysophosphatidylglycerol; LPS, Lysophosphatidylserine; PC, Phosphatidylcholine; PC-O, Alkylglycerophosphocholines; PE, Phosphatidylethanolamine;

PE-P, alkenylglycerophosphoethanolamines; PG, Phosphatidylglycerol; PS, Phosphatidylserine; SM, Sphingomyelin; TG, Triacylg-lycerol; BA, Bile Acid; FFA, Free fatty acid; Eicosanoid, Eicosanoids; LPI, Lysophosphatidylin-ositol; PI, Phosphatidylinositol; LPA, Lysophosphatidic a cid; PA, Phosphatidic acid; PMeOH, Phosphatidylmethanol; PE-O, alkylglycerophosphoethanol amin-es; BMP, Bismonoacylglyceropho-sphate; LNAPE, N-acyl-lysophosphatidylethanolamine; DG-O, Ether-linked diacylglycerol; SHexCer, Sulfatide; Cer-AP, Ceramide alpha-hydroxy fatty acid-phytospingosine; Cer-AS, Cerami-de alpha-hydroxy fatty acid-sphingosine; Cer-NDS, Cer amide non-hydroxyfatty acid-dihydrosp-hingosine; Cer-NP, Ceramide non-hydroxyfatty acid-ph ytospingosine; Cer-NS, Ceramide non-hy-droxyfatty acid-sphingosine; HexCer-AP, Hexosylcera mide alpha-hydroxy fatty acid-phytospin-gosine; HexCer-NS, Hexosylceramide non-hydroxyfatt y acid-sphingosine.

Supplementary Figures

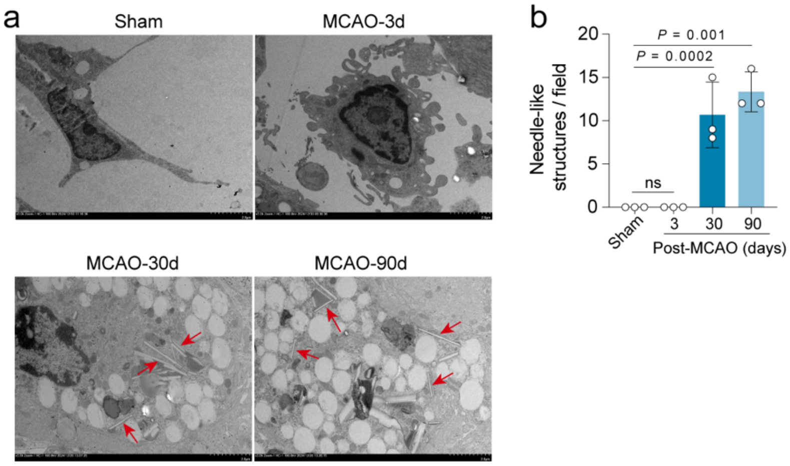

Supplementary Fig. 1 | Transmission electron microscopy (TEM) reveals needle-like structures in the ischemic brain during the chronic stage post-stroke

a, TEM images showing crystal structures (arrows) in the ischemic brain at days 30 and 90 post-MCAO. Scale bar, 2 μm.

b, Quantification of needle-like structures at different time points after MCAO. n = 3 mice/group. Statistical significance was assessed by one-way ANOVA (**b**) followed by Dunnett's multiple comparisons test.

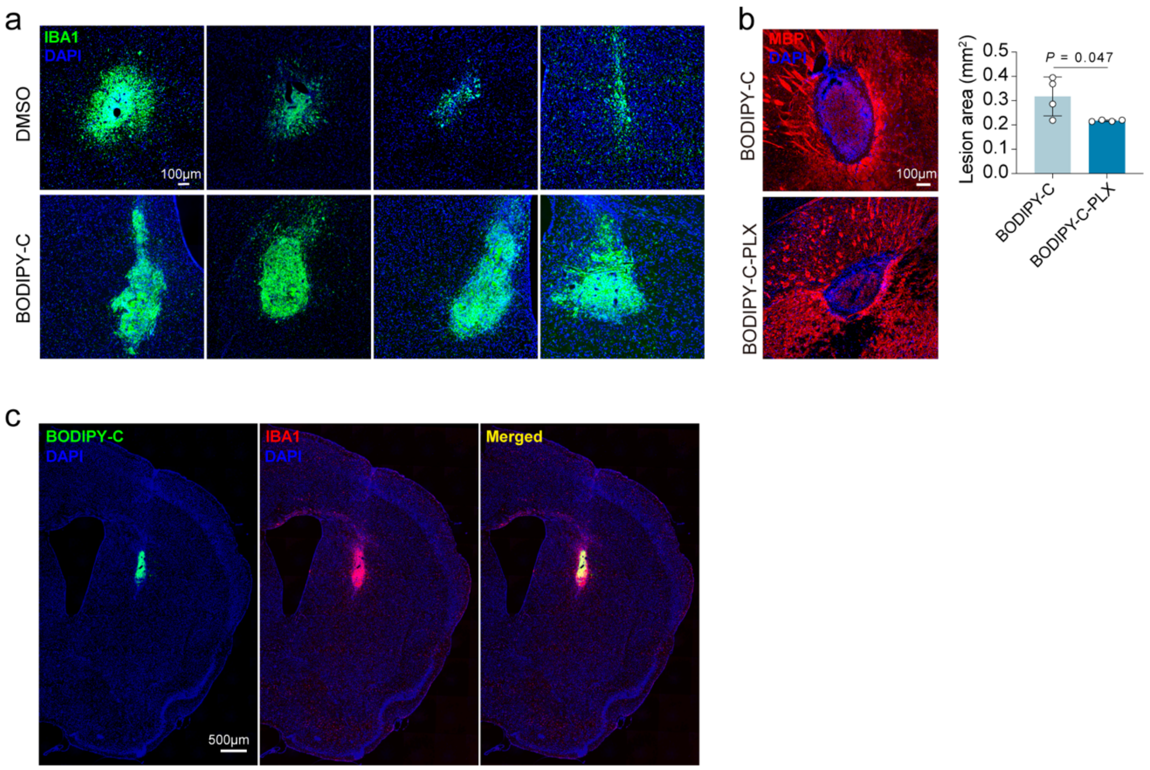

Supplementary Fig. 2 | BODIPY-Cholesterol overload induces microglial activation, while microglial depletion mitigates BODIPY–C–induced myelin injury

a, BODIPY-C injection induced increased microglial activation during the chronic stage post-injection, compared to DMSO-treated controls. Scale bar, 100 μm.

b, Myelin basic protein (MBP) immunostaining illustrating the effect of PLX5622 (PLX) or vehicle treatment on white matter repair following BODIPY-C injection. Microglial depletion with PLX mitigated BODIPY-C-induced myelin damage. Scale bar, 100 μm; n = 4 mice/group.

c, Visualization of the injection site using BODIPY-C fluorescence, with focal microglial activation identified by IBA1 staining. Scale bar, 500 μm.

Data are presented as mean ± SD. Statistical significance was assessed by a two-sided unpaired Student's t-test (**b**).

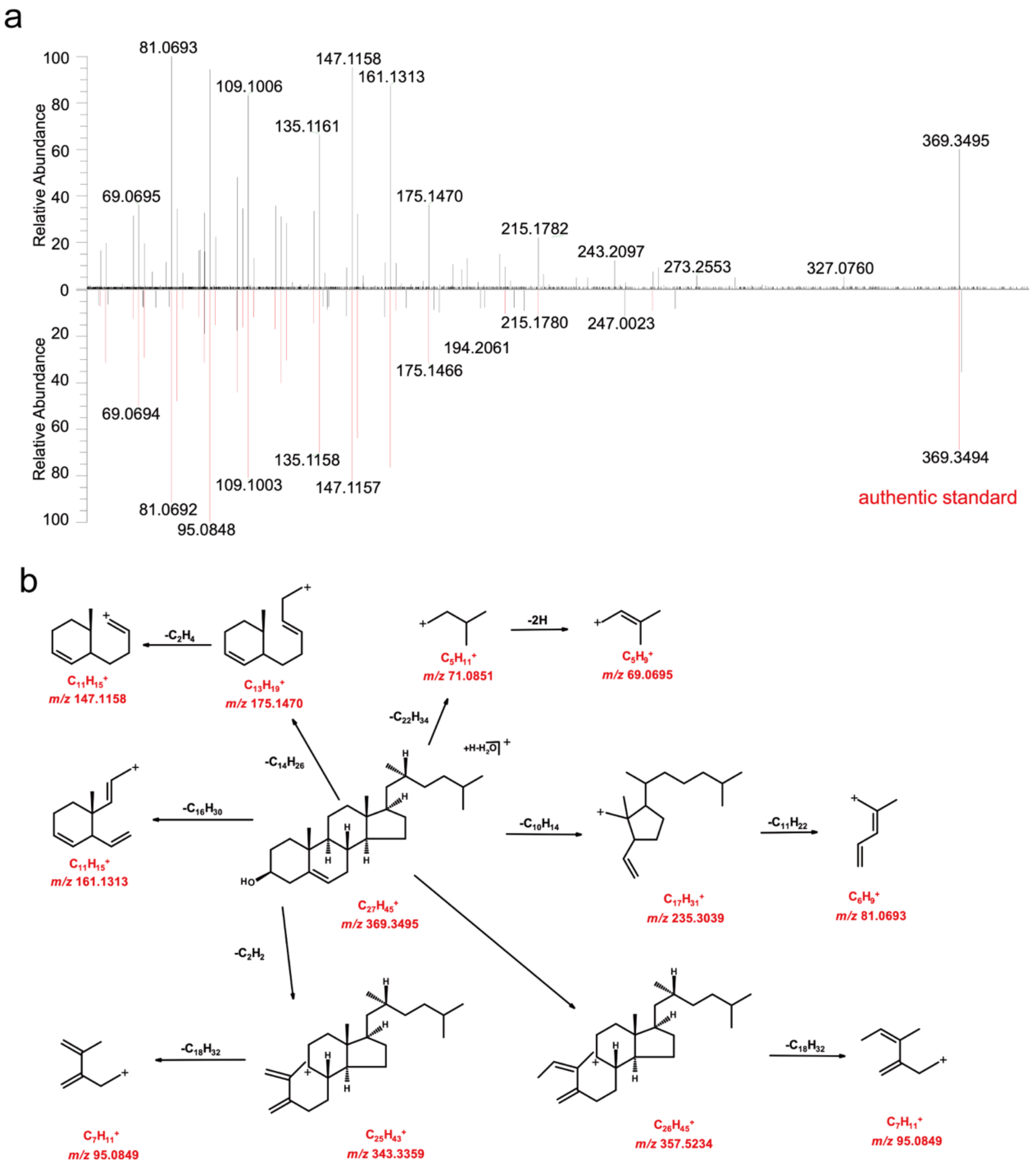

Supplementary Fig. 3 | The MS/MS spectrum and the fragmentation pathway of cholesterol

The MS/MS spectrum (**a**) and the fragmentation pathway (**b**) of cholesterol (m/z 369.3495 [M+H-H2O]+) in positive scan mode. The high-resolution MS and MS/MS spectra of cholesterol were compared with the standard.

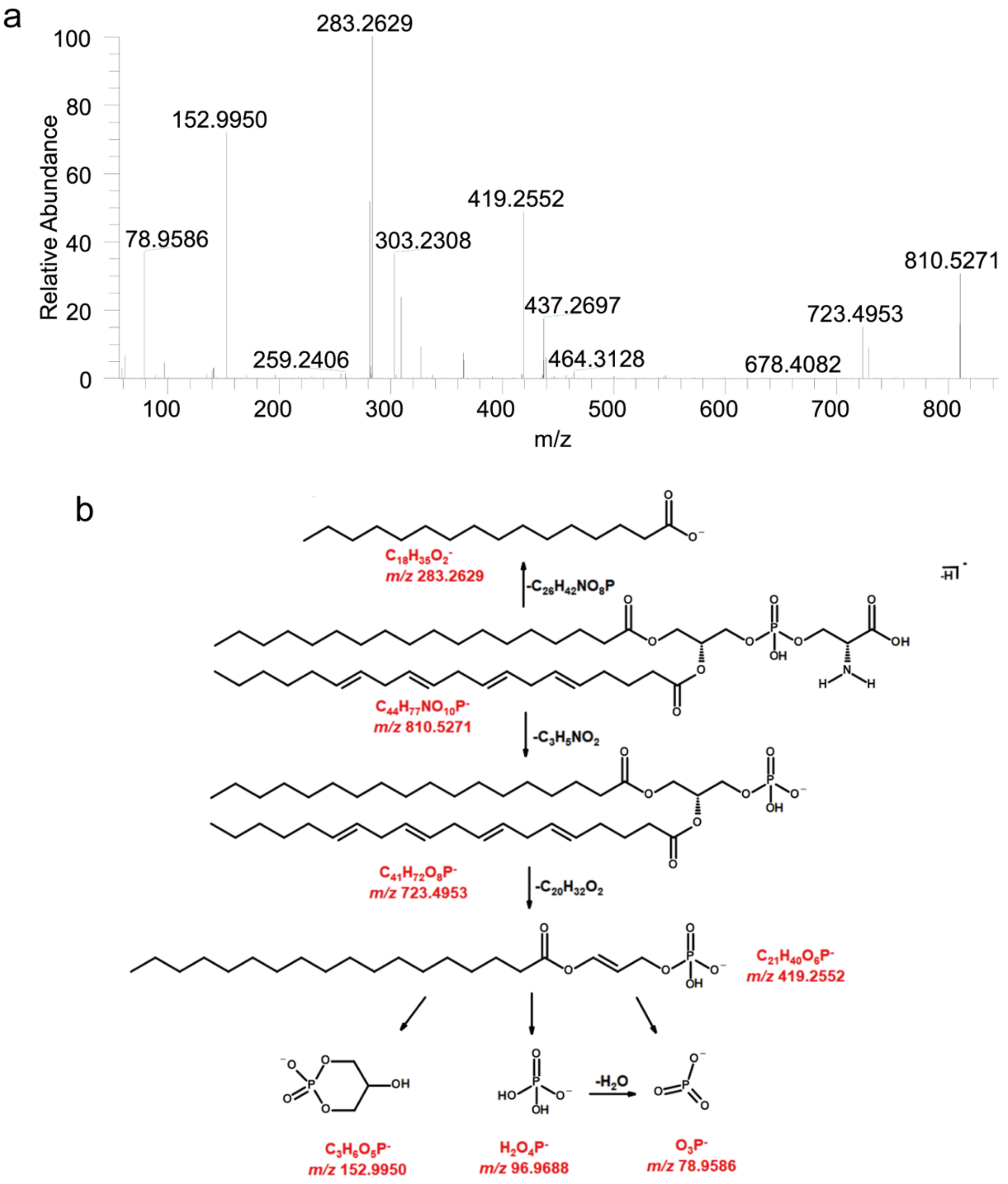

Supplementary Fig. 4 | The MS/MS spectrum and the fragmentation pathway of PS (18:0/20:4)

a, MS/MS spectrum of PS (18:0/20:4) (m/z 810.5271 [M+H]+) acquired in positive ion mode.

b, Proposed fragmentation pathway of PS (18:0/20:4) based on the observed MS/MS fragments.

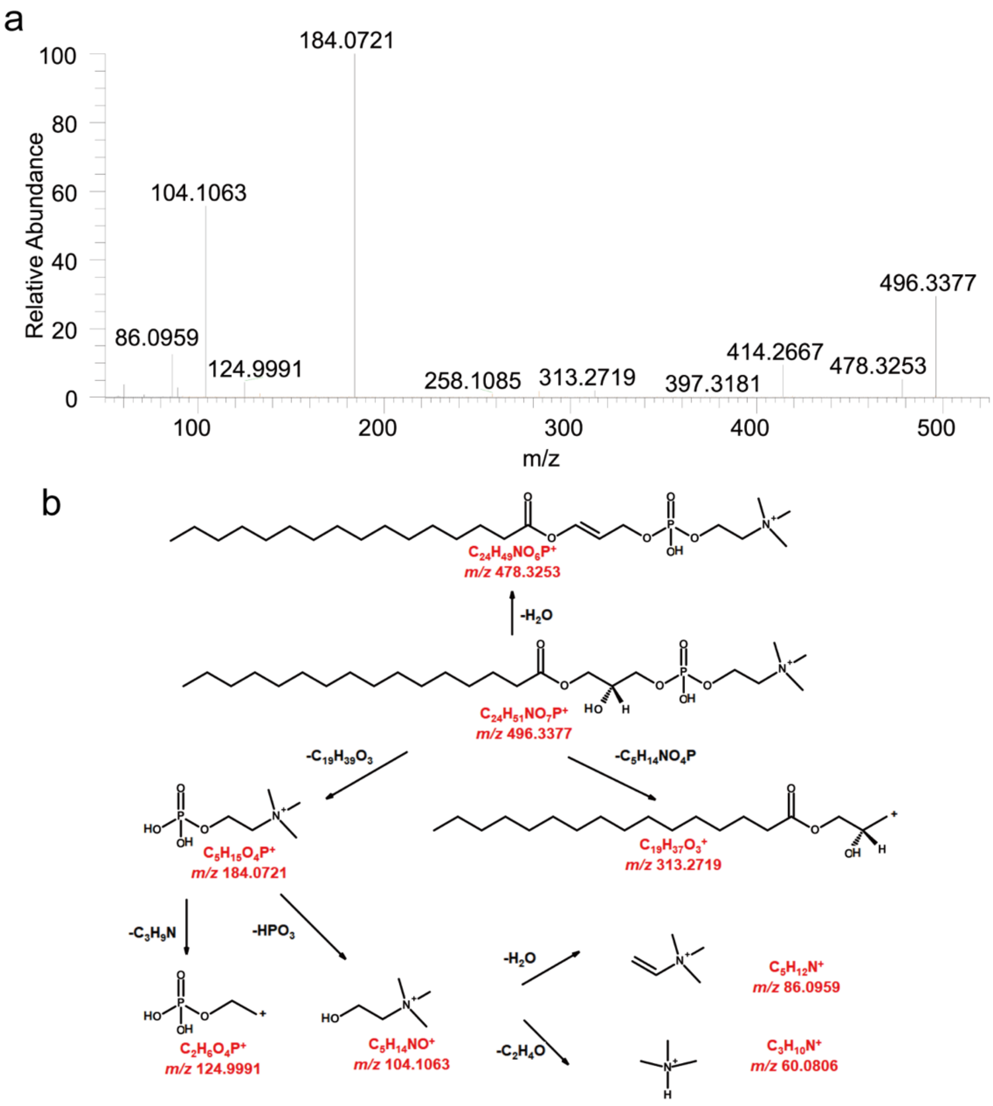

Supplementary Fig. 5 | The MS/MS spectrum and the fragmentation pathway of LPC (16:0)

The MS/MS spectrum (**a**) and the fragmentation pathway (**b**) of LPC (16:0) (m/z 496.3377 [M+H]+) in positive scan mode.

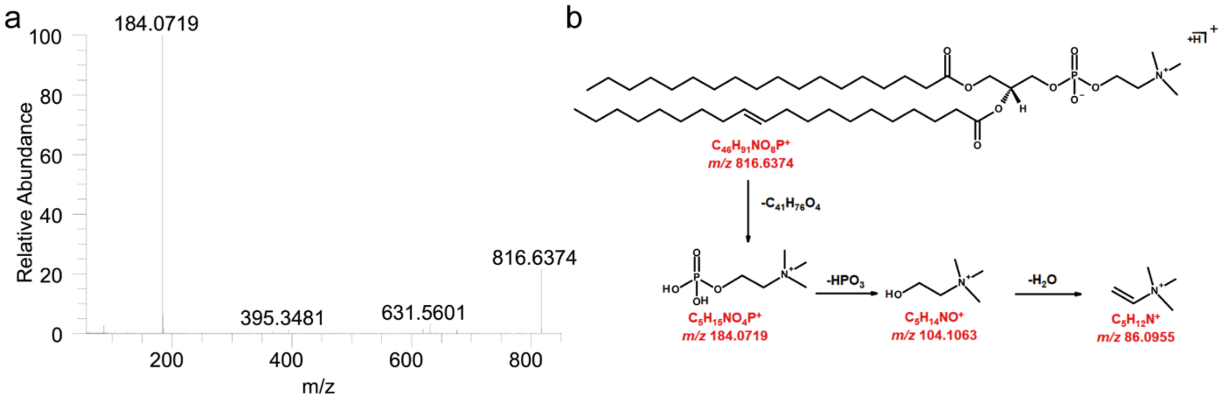

Supplementary Fig. 6 | The MS/MS spectrum and the fragmentation pathway of PC (18:0/20:1)

The MS/MS spectrum (**a**) and the fragmentation pathway (**b**) of PC (18:0/20:1) (m/z 816.6374 [M+H]+) in positive scan mode.

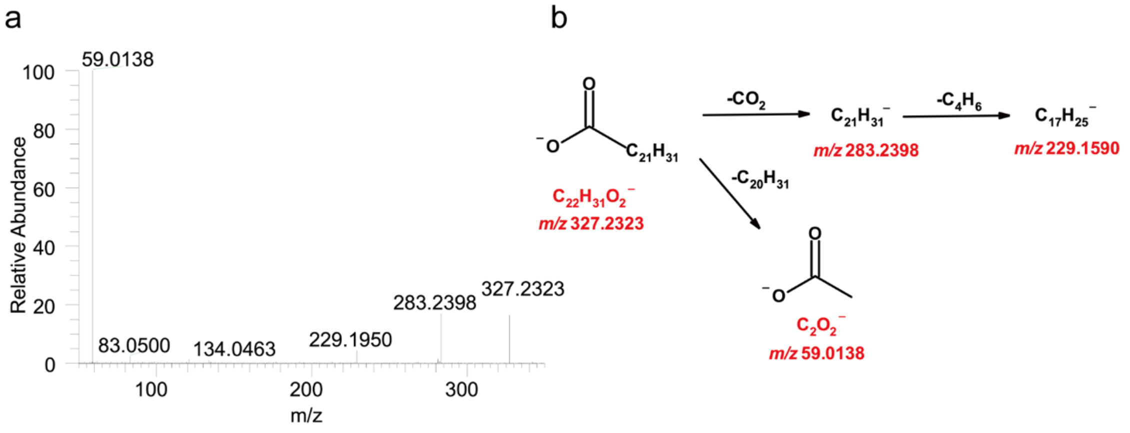

Supplementary Fig. 7 | The MS/MS spectrum and the fragmentation pathway of FA 20:6

The MS/MS spectrum (**a**) and the fragmentation pathway (**b**) of FA 20:6 (m/z 327.2323 [M-H]-) in negative scan mode.

Supplementary Tables

Supplementary Table 1 | Medical Animal Target Lipidomics: Internal Standard Information

Supplementary Table 2 | Changes in lipid species in $CD11^+$ microglia during the acute and chronic stage post-MCAO

Supplementary Table 3 | Changes in lipid species in serum during the chronic stage post-MCAO

Supplementary Table 4 | Changes in lipid species in cerebrospinal fluid (CSF) during the chronic stage post-MCAO
