# Supplementary material for: Cholesterol metabolic reprogramming mediates microglia-induced chronic neuroinflammation and hinders neurorestoration following stroke
Source: Nat Metab. 2025 Sep 23;7(10):2099–116. doi: 10.1038/s42255-025-01379-7 (PMC12552130; doi:10.1038/s42255-025-01379-7)
Supplement: Supplementary file 2 — Reporting Summary [file 42255_2025_1379_MOESM2_ESM.pdf]

nature portfolio

Corresponding author(s): Junwei Hao

Last updated by author(s): Jul 15, 2025

Reporting Summary

Nature Portfolio wishes to improve the reproducibility of the work that we publish. This form provides structure for consistency and transparency in reporting. For further information on Nature Portfolio policies, see our Editorial Policies and the Editorial Policy Checklist.

Statistics

For all statistical analyses, confirm that the following items are present in the figure legend, table legend, main text, or Methods section.

n/a	Confirmed	
☐	☒	The exact sample size (*n*) for each experimental group/condition, given as a discrete number and unit of measurement
☐	☒	A statement on whether measurements were taken from distinct samples or whether the same sample was measured repeatedly
☐	☒	The statistical test(s) used AND whether they are one- or two-sided<br>*Only common tests should be described solely by name; describe more complex techniques in the Methods section.*
☒	☐	A description of all covariates tested
☐	☒	A description of any assumptions or corrections, such as tests of normality and adjustment for multiple comparisons
☐	☒	A full description of the statistical parameters including central tendency (e.g. means) or other basic estimates (e.g. regression coefficient) AND variation (e.g. standard deviation) or associated estimates of uncertainty (e.g. confidence intervals)
☐	☒	For null hypothesis testing, the test statistic (e.g. *F*, *t*, *r*) with confidence intervals, effect sizes, degrees of freedom and *P* value noted<br>*Give P values as exact values whenever suitable.*
☒	☐	For Bayesian analysis, information on the choice of priors and Markov chain Monte Carlo settings
☒	☐	For hierarchical and complex designs, identification of the appropriate level for tests and full reporting of outcomes
☒	☐	Estimates of effect sizes (e.g. Cohen's *d*, Pearson's *r*), indicating how they were calculated

Our web collection on statistics for biologists contains articles on many of the points above.

Software and code

Policy information about availability of computer code

Data collection	Single-cell RNA sequencing was conducted by novogene using the Illumina platform.<br>The relative quantification of lipids was performed using a targeted lipidomics method via LC-MS/MS with multiple reaction monitoring.<br>Bulk RNA sequencing was conducted by Biomarker Technologies Co., Ltd.<br>Flow cytometry was performed using BD FACSAria™ III.<br>Infarct volume in MCAO-induced mice was analyzed using a 7T MRI scanner (Bruker Daltonics Inc., USA), with volumetric calculations performed via ITK-SNAP V4.2.2.<br>All fluorescence images were captured on confocal microscope (Zeiss LSM900) with ZEN 2.1 blue edition softwae.<br>Cholesterol crystals were visualized using Polarized Light Microscopy (PLM) with crossed polarizers on Zeiss LSM900 or/and Zeiss Imager. M2 microscope.<br>Whole brain images were acquired by the Mica microscope(Leica).<br>The JEM 2100 transmission electron microscope was used to assess the ultrastructure of microglia and myelin sheath.
Data analysis	Single-cell RNA sequencing data processing softwares used are as follow: CellRanger V7.0.0, Seurat package V3.1.1, and Monocle V3.<br>Gene Ontology (GO) enrichment analysis of the differentially expressed genes (DEGs) was implemented by the clusterProfiler packages.<br>KOBAS database and clusterProfiler software were employed to test the statistical enrichment of differential expression genes in KEGG pathways.<br>The analysis of lipidomics was carried out using an ExionLC UHPLC system coupled with a QTRAP 6500+ Mass Spectrometer (AB SCIEX LLC, Framingham, USA), controlled by Analyst 1.6.3 software (AB SCIEX).<br>For quantification of the immunofluoresence signals, we used ImageJ 1.54g software (NIH, Bethesda, MD).<br>Whole brain images were analyzed by the Leica Application Suite X.<br>BD FACSDiva™ Software (V8.0) was employed to analyse the data from flow cytometry.

The MRI data were analyzed using ITK-SNAP software V4.2.2 (National Institutes of Health, Bethesda, MD, USA).
Statistical analyses were performed using GraphPad Prism (V10.2.3) software.
For two-group comparisons, unpaired two-tailed Student's t-tests were used. For three or more groups, one-way ANOVA followed by Dunnett's or Bonferroni's multiple comparisons test was applied. When two independent variables were involved, two-way ANOVA with Bonferroni's multiple comparisons test was used. P<0.05 was considered statistically significant.

For manuscripts utilizing custom algorithms or software that are central to the research but not yet described in published literature, software must be made available to editors and reviewers. We strongly encourage code deposition in a community repository (e.g. GitHub). See the Nature Portfolio guidelines for submitting code & software for further information.

Data

Policy information about availability of data

All manuscripts must include a data availability statement. This statement should provide the following information, where applicable:

- Accession codes, unique identifiers, or web links for publicly available datasets
- A description of any restrictions on data availability
- For clinical datasets or third party data, please ensure that the statement adheres to our policy

The raw data for scRNA-seq and bulk RNA-seq reported in this paper have been deposited in the Genome Sequence Archive (GSA) and are publicly available under accession code CRA017588 and CRA017573. Quantitative data that support the findings of this study are available within the paper. Extended Data are contained in the main text. Source data are provided with this paper.

Research involving human participants, their data, or biological material

Policy information about studies with human participants or human data. See also policy information about sex, gender (identity/presentation), and sexual orientation and race, ethnicity and racism.

Reporting on sex and gender	None
Reporting on race, ethnicity, or other socially relevant groupings	None
Population characteristics	None
Recruitment	None
Ethics oversight	None

Note that full information on the approval of the study protocol must also be provided in the manuscript.

Field-specific reporting

Please select the one below that is the best fit for your research. If you are not sure, read the appropriate sections before making your selection.

☒ Life sciences ☐ Behavioural & social sciences ☐ Ecological, evolutionary & environmental sciences

For a reference copy of the document with all sections, see nature.com/documents/nr-reporting-summary-flat.pdf

Life sciences study design

All studies must disclose on these points even when the disclosure is negative.

Sample size	Sample sizes are indicated for each experiment in corresponding figures by showing individual points for each replicate/animal. Sample size for all experiments including expression analysis where chosen based on statistical requirements which is in line with most published data and own experiences. The number of animals used for MCAO experiments is similar to those in other studies and was based on previous experience with these models in our laboratory.
Data exclusions	No data were excluded
Replication	All experimental findings were reproduced in multiple independent experiments. The number of independent experiments and biological replicates in each data panel is indicated in the figure legends.
Randomization	Mice were assigned randomly into experimental groups and processed in an arbitrary order.
Blinding	All mice and subsequent samples were labelled only with mouse ID numbers and did not indicate genotype or type of treatment. Genotype or treatment type were decoded after the data acquisition and quantification analysis were complete.

Reporting for specific materials, systems and methods

We require information from authors about some types of materials, experimental systems and methods used in many studies. Here, indicate whether each material, system or method listed is relevant to your study. If you are not sure if a list item applies to your research, read the appropriate section before selecting a response.

Materials & experimental systems

n/a	Involved in the study
☐	☒ Antibodies
☒	☐ Eukaryotic cell lines
☒	☐ Palaeontology and archaeology
☐	☒ Animals and other organisms
☒	☐ Clinical data
☒	☐ Dual use research of concern
☒	☐ Plants

Methods

n/a	Involved in the study
☒	☐ ChIP-seq
☐	☒ Flow cytometry
☐	☒ MRI-based neuroimaging

Antibodies

Antibodies used

rabbit anti-Iba1 (1:1000, 019-19741,Wako)
rabbit anti-Myelin basic protein (1:500, ab7349, Abcam)
rabbit anti-cyp46a1(1:100, 12486-1-AP, Proteintech)
donkey anti-rabbit IgG Alexa Fluor 568 (1:1000, A78946, Thermo Fisher)
donkey anti-rabbit IgG Alexa Fluor 647 (1:1000, A31573, Thermo Fisher)
donkey anti-rat IgG Alexa Fluor 647 (1:1000, A78947, Thermo Fisher)
donkey anti-rabbit IgG Alexa Fluor 488 (1:1000, A21206, Thermo Fisher)
donkey anti-rat IgG Alexa Fluor 488 (1:1000, A21208, Thermo Fisher)
PE/Cyanine7 anti-mouse CD45 Antibody(1:100, 103114, BioLegend)
PE anti-mouse/human CD11b Antibody (1:100, 101208, BioLegend)
Brilliant Violet 605™ anti-mouse F4/80 Antibody(1:100, 123133, BioLegend)
APC anti-mouse ACSA-2 Antibody (1:100, 130-117-535, Miltenyi Biotec)

Validation

All antibodies were validated for the indicated species and applications by manufacturer's references and/or supporting literature.

Animals and other research organisms

Policy information about studies involving animals; ARRIVE guidelines recommended for reporting animal research, and Sex and Gender in Research

Laboratory animals

Adult male C57BL/6J mice (8-10 weeks old) were obtained from Beijing Vital River Laboratory Animal Technology. R26-LSL-Cyp46a1 mice were purchased from Shanghai Model Organisms Center, Inc. Cx3cr1-CreER-EYFP mice were purchased from The Jackson Laboratory. All mice were housed in a SPF facility at 24 ± 2°C and 60 ± 5% relative humidity under a 12-h light-dark cycle with food and water available ad libitum. Adequate measures were taken to minimize the number of animals used and ensure minimal pain or discomfort.

Wild animals

none

Reporting on sex

Male and female

Field-collected samples

none

Ethics oversight

Experimental protocols were approved by the Xuanwu Hospital Capital Medical University Animal Care and Use Committee.This study was conducted in accordance with the National Institutes of Health guidelines for the use of experimental animals.

Note that full information on the approval of the study protocol must also be provided in the manuscript.

Plants

Seed stocks: none

Novel plant genotypes: none

Authentication: none

Flow Cytometry

Plots

Confirm that:

- [x] The axis labels state the marker and fluorochrome used (e.g. CD4-FITC).
- [x] The axis scales are clearly visible. Include numbers along axes only for bottom left plot of group (a 'group' is an analysis of identical markers).
- [x] All plots are contour plots with outliers or pseudocolor plots.
- [x] A numerical value for number of cells or percentage (with statistics) is provided.

Methodology

Sample preparation: Mice were sacrificed under deep ketamine/xylazine anesthesia. Then, mice were infused with ice-cold 1×PBS (C3580-0500, VivaCell), followed closely by separating and collecting brain samples with the forebrain and cerebellum dissected from mice. Brain tissue was cut into pieces and digested with collagenase D (1 mg/ml, Roche Diagnostics) at 37 °C for 50 minutes. Afterward, digested tissue was passed through a 70-µm cell strainer (BD Falcon). The parenchyma was centrifuged on the 30% Percoll (17089109, Cytiva) gradient to remove myelin. The interphase was removed, washed, and resuspended with ice-cold 1×PBS with 0.5% Bovine Serum Albumin (BSA, V900933, Sigma-Aldrich). Then, we add 10 µL CD11b MicroBeads (30093634, Miltenyi Biotec) to per 10^7 total cells for 15 minutes, followed by adding 10 µL of CD11b-FITC (101206, BioLegend) and incubating for 5 minutes in the 4 °C refrigerator to assess the purity of cell.

Instrument: BD FACSAria™ III

Software: BD FACSDiva™ Software

Cell population abundance: microglia were identified by CD11b+ cells

Gating strategy: FSC-A and SSC-A were used to identify the starting cell population. FSC-A and FSC-H were used to identify the single cell subpopulation. FSC-A and 7-AAD-low were used to identify the live cell subpopulation. SSC-A and CD11b-high were used to identify the astrocyte subpopulation.

- [ ] Tick this box to confirm that a figure exemplifying the gating strategy is provided in the Supplementary Information.

Magnetic resonance imaging

Experimental design

Design type: NA

Design specifications: MRI T2 data were obtained from mice with MCAO surgery.

Behavioral performance measures: NA

Acquisition

Imaging type(s): T2-weighted images

Field strength: 7 Tesla

Sequence & imaging parameters: The animal’s respiration was continually monitored by a small animal monitoring and gating system (SA Instruments, Stoney Brook, NY, USA) via a pillow sensor positioned under the abdomen. Mice were placed on a heated circulating water blanket (Bruker, Billerica, MA) to maintain average body temperature (37 °C). Axial 2D multi-slice T2-weighted

images of the brain with fat-suppressed Rapid Acquisition with Relaxation Enhancement (RARE) sequence (TR = 4000 ms, effective TE = 60 ms, number of averages = 4, FOV = 19.2 mm × 19.2 mm, matrix size = 192 × 192). The 25 gradient echoes were acquired for T2-mapping using a Multislice Gradient Echo sequence.

Area of acquisition: Whole brain

Diffusion MRI: ☐ Used ☒ Not used

Preprocessing

Preprocessing software: The MRI data were analyzed using ITK-SNAP software (National Institutes of Health, Bethesda, MD, USA).

Normalization: None

Normalization template: None

Noise and artifact removal: None

Volume censoring: None

Statistical modeling & inference

Model type and settings: None

Effect(s) tested: None

Specify type of analysis: ☐ Whole brain ☐ ROI-based ☒ Both

Anatomical location(s): *Describe how anatomical locations were determined (e.g. specify whether automated labeling algorithms or probabilistic atlases were used).*

Statistic type for inference: None

(See Eklund et al. 2016)

Correction: None

Models & analysis

n/a	Involved in the study
☒	☐ Functional and/or effective connectivity
☒	☐ Graph analysis
☒	☐ Multivariate modeling or predictive analysis
